# Supplementary material for: Hypersensitive meta-crack strain sensor for real-time biomedical monitoring
Source: Sci Adv. 2024 Dec 20;10(51):eads9258. doi: 10.1126/sciadv.ads9258 (PMC11661431; doi:10.1126/sciadv.ads9258)
Supplement: 20241220-1 [file sciadv.ads9258.v1.pdf]

PHYSICAL SCIENCES

Hypersensitive meta-crack strain sensor for real-time biomedical monitoring

Jae-Hwan Lee[1]†, Yoon-Nam Kim[1]†, Junsang Lee[2]†, Jooik Jeon[3], Jae-Young Bae[1], Ju-Yong Lee[1], Kyung-Sub Kim[1], Minseong Chae[1,4], Hyunjun Park[5], Jong-hyoung Kim[6], Kang-Sik Lee[4], Jeonghyun Kim[7], Jung Keun Hyun[3,8,9]*, Daeshik Kang[10]*, Seung-Kyun Kang[1,11,12]*

Copyright © 2024 The Authors, some rights reserved; exclusive licensee American Association for the Advancement of Science. No claim to original U.S. Government Works. Distributed under a Creative Commons Attribution License 4.0 (CC BY).

Real-time monitoring of infinitesimal deformations on complex morphologies is essential for precision biomechanical engineering. While flexible strain sensors facilitate real-time monitoring with shape-adaptive properties, their sensitivity is generally lower than spectroscopic imaging methods. Crack-based strain sensors achieve enhanced sensitivity with gauge factors (GFs) exceeding 30,000; however, such GFs are only attainable at large strains exceeding several percent and decline below 10 for strains under 10^{-3}, rendering them inadequate for minute deformations. Here, we introduce hypersensitive and flexible "meta-crack" sensors detecting infinitesimal strains through previously undiscovered crack-opening mechanisms. These sensors achieve remarkable GFs surpassing 1000 at strains of 10^{-4} on substrates with a Poisson's ratio of −0.9. The crack orientation–independent gap-widening behavior elucidates the origin of hypersensitivity, corroborated by simplified models and finite element analysis. Additionally, parallel mechanical circuits of meta-cracks effectively address the trade-off between resolution and maximum sensing threshold. In vivo real-time monitoring of cerebrovascular dynamics with a strain resolution of 10^{-5} underscores the hypersensitivity and conformal adaptability of sensors.

INTRODUCTION

The continuous assessment of small-scale deformations on soft and complex morphologies is becoming crucial in precise biomechanical engineering including mechanobiology (*1*–*3*), diagnostic biomedicine (*4*, *5*), health care monitoring (*6*–*8*), and human behavioral biometrics (*9*–*11*). The ability to measure previously undetectable, extremely infinitesimal strains unlocks previously unexplored possibilities, such as monitoring intracellular tension dynamics at the single-cell level during metabolic processes (*2*, *3*, *12*), facilitating the early diagnosis of diseases associated with cerebrovascular and hemodynamic responses (*7*, *13*), identifying previously undiscovered mechano-biomarkers (*14*–*16*), and enabling remote monitoring of deep-body mechanical phenomena (*17*–*19*). In bio-interfaced environments, flexible strain sensors (*6*, *9*, *20*–*25*) serve as immediate mechano-responsive indicators, using electrical (*6*, *9*, *20*, *21*), optical (*22*, *23*), and magnetic (*24*, *25*) signals to support continuous and long-term monitoring. These sensors present notable advantages over traditional spectroscopic imaging techniques (*1*–*3*, *26*), which require complex data processing, by providing real-time, continuous monitoring capabilities. Moreover, their exceptional adaptability to diverse surfaces minimizes damage to biological targets (*6*, *9*) and enables accurate detection (*1*, *7*, *27*) through conformal contact based on mechanical compliance (*27*, *28*).

Flexible and soft strain sensors, however, often exhibit limited strain sensitivity with GFs typically below 1000 (*6*, *21*–*24*), thereby restricting their application to relatively large deformations, such as physiological strains occurring on the skin, blood vessels, or other organs. Recent advancements (*29*–*32*) have demonstrated that incorporating nanoscale cracks into thin conductive films (*33*) can remarkably enhance sensitivity, achieving ultrahigh strain sensitivity with GFs exceeding 10,000. This enhancement is primarily due to the localized deformation at the crack sites, which induces substantial changes in electrical resistance through the disconnection of crack edges. Further strategies to increase sensitivity, achieving GFs beyond 30,000, involve optimizing crack depth and density (*30*, *34*), precisely controlling the thickness of the conductive layer (*35*, *36*), and applying preset tension during sensor fabrication (*31*, *32*).

However, the realization of such extraordinarily high GFs is confined to relatively large strain regimes, often exceeding several percent. At infinitesimal strains below 10^{-3}, the GF markedly diminishes, even falling below the performance of silicon strain gauges, which can achieve values up to 100 (*21*, *33*). This reduction in sensitivity is attributed to the reconnection of crack edges induced by Poisson compression strain during the initial phase of crack opening. As a result, the minimum detectable strain for crack-based sensors is constrained to the order of 10^{-3} (*31*), despite the common perception of their high sensitivity. Furthermore, the resistance of these sensors increases exponentially due to crack opening, rapidly approaching electrical disconnection and thus defining the sensing limit at strains below a few percent (*33*). The excessive resistance variation prevents accurate detection of strains beyond several tens of percent, highlighting the trade-off between strain resolution and maximum sensing threshold.

[1]Department of Materials Science and Engineering, Seoul National University, Seoul 08826, Republic of Korea. [2]Weldon School of Biomedical Engineering, Purdue University, West Lafayette, IN 47907, USA. [3]Department of Nanobiomedical Science and BK21 NBM Global Research Center for Regenerative Medicine, Dankook University, Cheonan 31116, Republic of Korea. [4]Biomedical Engineering Research Center, Asan Medical Center, Seoul 05505, Republic of Korea. [5]Department of Chemical Engineering, Kwangwoon University, Seoul 01897, Republic of Korea. [6]Department of Materials Science and Engineering, Pukyong National University, Busan 48513, Republic of Korea. [7]Department of Electronic Convergence Engineering, Kwangwoon University, Seoul 01897, Republic of Korea. [8]Department of Rehabilitation Medicine, College of Medicine, Dankook University, Cheonan 31116, Republic of Korea. [9]Institute of Tissue Regeneration Engineering (ITREN), Dankook University, Cheonan 31116, Republic of Korea. [10]Department of Mechanical Engineering, Ajou University, Suwon 16499, Republic of Korea. [11]Research Institute of Advanced Materials (RIAM), Seoul National University, Seoul 08826, Republic of Korea. [12]Nano Systems Institute SOFT Foundry, Seoul National University, Seoul 08826, Republic of Korea.
*Corresponding author. Email: kskg7227@snu.ac.kr (S.-K.K.); dskang@ajou.ac.kr (D.K.); rhhyun@dankook.ac.kr (J.K.H.)
†These authors contributed equally to this work.

Here, we introduce a "meta-crack behavior" in the thin metal film of a flexible strain sensor, which remarkably enhances strain sensitivity even under infinitesimal strains on the order of 10^{-5}. This improvement is facilitated by a mechanical meta-substrate with a negative Poisson's ratio, enabling unusual crack opening by minimizing contact at the crack edges through the manipulation of local sweeping phenomena. Validation of the meta-crack opening was achieved through scanning electron microscope (SEM) imaging and finite element analysis (FEA). We overcome the intrinsic trade-off between strain resolution and maximum sensing threshold by exploiting parallel mechanical circuits of meta-cracks. The parallel-based unique arrangement of electrical pathways mitigates the resistance spike beyond the critical strain, achieving a 10^{-5} scale strain resolution while simultaneously maintaining the maximum sensing limit comparable to that of conventional crack sensors. In vivo demonstrations verified the real-time continuous monitoring of previously undetectable microvascular dynamics on the cerebral cortex. Monitoring of blood flux and specific pulsatile patterns in branches of the cerebral artery and superior cerebral vein demonstrated potential for real-time diagnosis of hemorrhagic or ischemic cerebrovascular and cardiovascular diseases. This involved the integration of a simple near-field communication (NFC)–based chipset for battery-free on-board monitoring.

RESULTS

Overview of a meta-crack for hypersensitive strain monitoring

Figure 1 describes the structure and working principle of a meta-crack exploiting Poisson's ratio–controlled crack opening for a hypersensitive strain sensor. Figure 1A shows an exploded view of the meta-crack sensor consisting of a crack-induced conductive layer (Mo, 50 nm thick), an adhesive layer (MoO_3, 15 nm thick), and an auxetic structure–embedded substrate [base matrix: poly-butylene adipate terephthalate (PBAT), 30 μm thick; auxetic structure: polylactic acid (PLA), 10 μm thick]. Figure S1 shows images of the sensor and magnified structures of Mo cracks and PLA re-entrant auxetic structures with a Poisson's ratio of −0.9 (the experimental value obtained by averaging the ratios within the range of 0 to 2×10^{-2} strain). Cyclic tensile stretching up to 2×10^{-2} strain generates regular interval cracks on the Mo thin film under the same conditions reported in a previous study (*35*). The PLA auxetic structure, patterned by focused ultraviolet (UV) laser, induces biaxial stretching in the PBAT/PLA substrate, causing the Mo cracks on top to stretch biaxially in response.

Figure 1 (B and C) shows the remarkably enhanced sensitivity, and Fig. 1 (D and E) describes different crack openings depending on the Poisson's ratio of the substrate. In conventional crack opening, exerted strain applied to the substrate generates inward lateral strain due to a positive Poisson's ratio, delaying the complete detachment of the crack edges and causing a slow increase in electrical resistance at small strains (Fig. 1D). (*33*) In meta-crack opening, the negative Poisson's ratio induces outward lateral strain, consequently implementing the biaxial stretching of the Mo crack and immediately opening the crack gap (Fig. 1E). SEM images verify the immediate crack gap opening by implementing the negative Poisson's ratio, leading to complete disconnection at an applied strain of 5×10^{-3}, while the positive Poisson's ratio maintains the crack edge contact under the same applied strain. The early crack opening causes a rapid increase in electrical resistance, giving hypersensitivity in an extremely small strain range. The meta-crack sensor (ν = −0.9) shows at least fourfold higher GF at a strain of 3×10^{-3} compared to conventional crack sensors with an unprecedented 10^{-5} scale strain resolution (Fig. 1B, details in table S1). Here, the voltage-controlled PZT piezoelectric linear actuator, with a tensile displacement rate of 127 nm/V, applied strain to the sensor in multiples of 7.8×10^{-5}, showing 10^{-3} scale normalized resistance variation with an SD of around 10^{-5}, demonstrating a strain resolution of 10^{-5} scale. The finely controlled environmental factors include temperature (<0.03°C), vibration (<0.05 mm), and air flow rate (<0.001 m/s), resulting in a normalized resistance noise SD of 0.00011 (Fig. 1C, details in text S1 and figs. S2 to S5).

The crack opening behavior of meta-cracks is further investigated for a comprehensive understanding of crack gap widening dependency on the Poisson's ratio and crack morphology using a simplified model of single orientation crack with FEA and experiments of crack gap widening simulation. Figure 1 (F and G) and fig. S6 show the crack gap widening behavior under 0 to 2×10^{-2} strain range with diverse Poisson's ratios. The crack gap widening rate progressively decreases as the crack orientation becomes increasingly parallel to the tensile direction (high θ value) under a Poisson's ratio of 0.3. The gap even narrows when the crack orientation becomes nearly horizontal to the tensile direction (θ of more than 70°). The crack gap widening rate under a Poisson's ratio of −0.9, in contrast, remains positive, showing a slight decreasing tendency up to a θ of 70° before becoming saturated (details in text S2). The results indicate that an arbitrarily shaped meta-crack, formed by a continuous connection of diverse orientations, would exhibit persistent gap widening in all locations, while this behavior would be observed only at limited locations with specific morphologies in the conventional crack. This leads to notable changes in electrical resistance under extremely small strains regardless of the crack morphology.

Tunable strain sensitivity using integration of meta-structures

The performance of the meta-crack sensor exhibits wide tunability in strain sensitivity attributed to the controllable Poisson's ratio of the substrate, resulting from the variation in crack opening behavior. Figure 2 (A and B) presents optical images and experimental Poisson's ratios (n = 10) within a 2×10^{-2} strain range for a plane (ν = 0.3) substrate and for each of the rotachiral (ν = −0.2), vertical re-entrant (ν = −0.5), and horizontal re-entrant (ν = −0.9) auxetic structured substrates. These substrates have specific geometries designed to achieve negative Poisson's ratios (here, Poisson's ratio is denoted by an average value ranging from 0 to 2×10^{-2} strain). Figure S7 provides unit cell geometries of the auxetic structure for FEA, calculating each Poisson's ratio within the same strain range. Figure S8 shows periodically uniform distribution of x- and y-axis strains and calculated Poisson's ratios across the meta-substrate surfaces based on FEA, dependent on each unit auxetic structure. The experimental Poisson's ratios were closely fit with the FEA results, yielding small error bars, when the measurement area matched the unit area of each auxetic structure and sufficient measurements were taken. The consistent FEA results with the experimental values are shown in Fig. 2B (line). The Poisson's ratio of the meta-substrate would also be influenced by the differences in stiffness between the

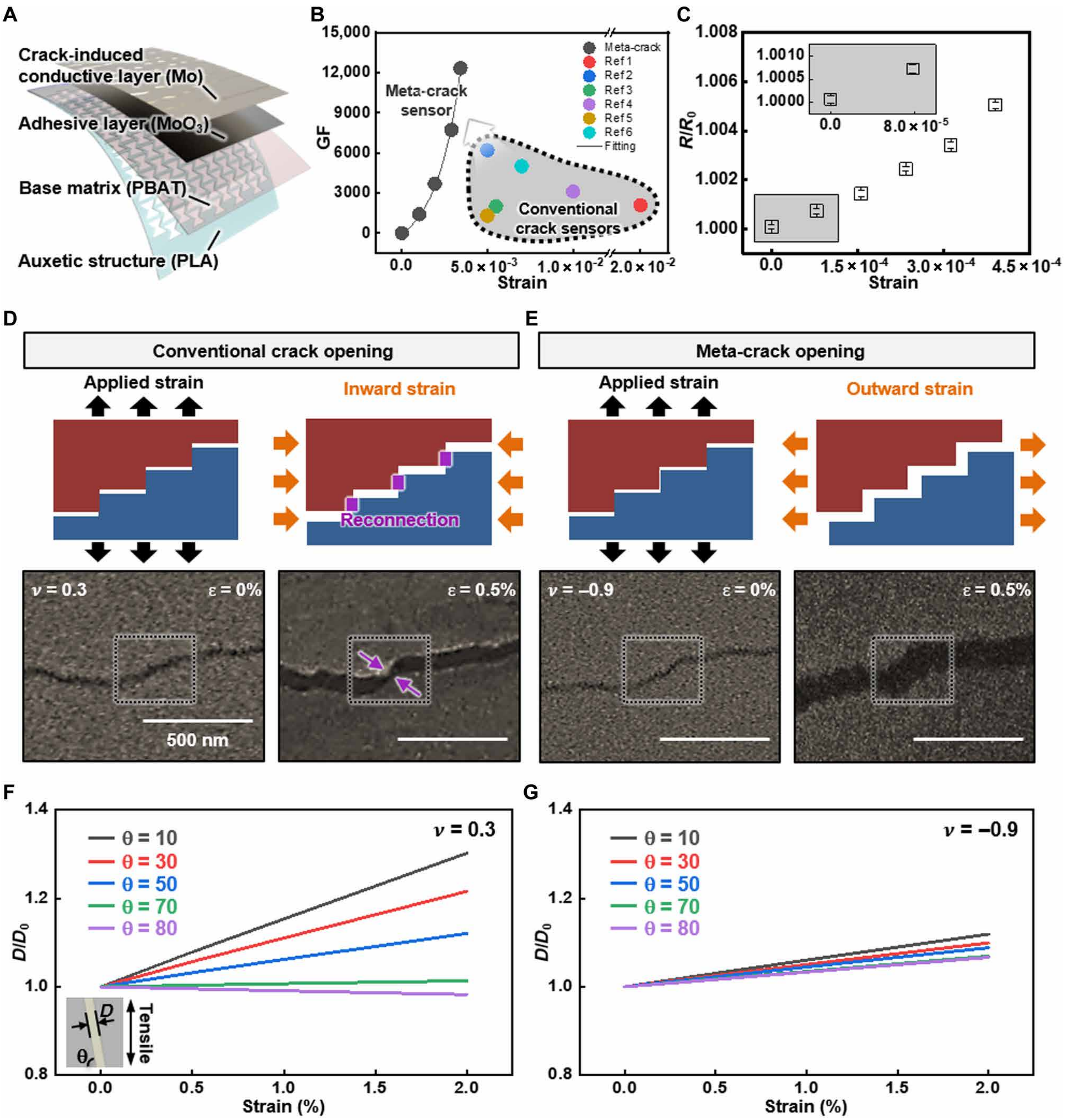

Fig. 1. A hypersensitive meta-crack for minute strain monitoring. (**A**) An exploded view illustration of the meta-crack integrated flexible hypersensitive strain sensor consists of crack-induced Mo, adhesive MoO_3, base PBAT substrate, and Poisson's ratio-controllable PLA auxetic structure. (**B**) Comparison of strain sensitivity of various previous crack sensors and the meta-crack sensor with a fitting line. (**C**) Strain sensitivity of the meta-crack sensor within extremely low strain range below the 10^{-4} scale. (**D** and **E**) Illustration and SEM images of the step-shaped crack boundary and their opening procedure under vertical tensile strain (5×10^{-3}) for conventional crack and meta-crack, controlled by localized Poisson's ratios of the substrates. Inward strain and corresponding reconnection of crack edges occur on conventional crack opening with positive Poisson's ratio (0.3), and outward strain and immediate disconnection occur on meta-crack opening with negative Poisson's ratio (−0.9). (**F** and **G**) Confirmation of crack gap widening behavior with crack orientation of 10°, 30°, 50°, 70°, and 80° under positive Poisson's ratio (0.3) and negative Poisson's ratio (−0.9) based on FEA. The normalized crack gap distance (D/D_0) shows continuously increasing states in all orientations under −0.9 Poisson's ratio, while presenting the opening behavior in specific orientations under 0.3 Poisson's ratio.

composing layers, and the buckling effect induced by the asymmetrical composition (details in figs. S9 to S13 and texts S3 and S4). Figure 2C shows the tunability of strain sensitivity with different Poisson's ratios. The GFs of the sensor with Poisson's ratios of −0.9, −0.5, −0.2, and 0.3 were 1450, 590, 130, and 10 at 10^{-4} strain, and 7720, 680, 270, and 30 at 2.9×10^{-3} strain, respectively (enlarged GF variation for the 10^{-4} scale strain range in fig. S14). The GF and Poisson's ratio have an inverse relationship, indicating the tunability of strain sensitivity through the control of the crack disconnection rate.

Figure 2D shows the normalized resistance of the sensors. The strain-resistance behavior under various Poisson's ratios can be

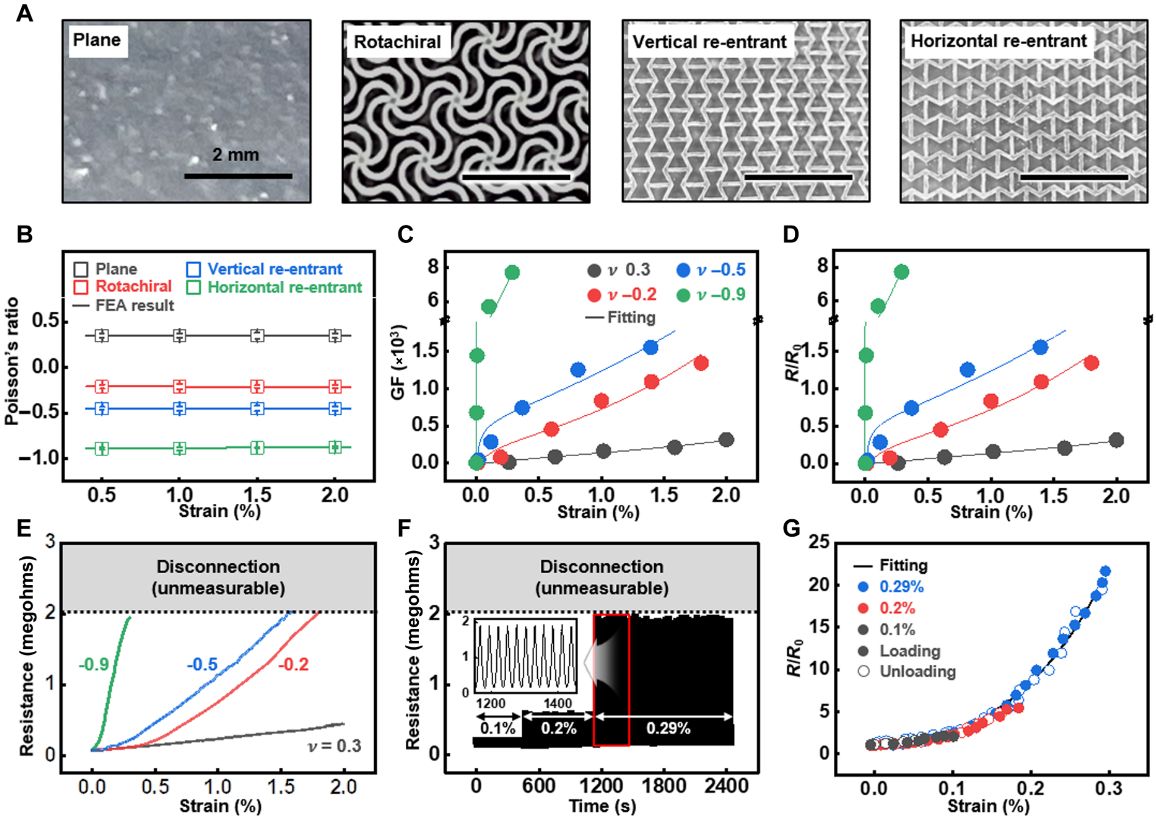

Fig. 2. Tuning of the sensitivity of meta-crack sensor with the integration of various substrates having different negative Poisson's ratios. (**A**) Optical images of plane substrate and diverse auxetic structures for different negative Poisson's ratios with specific geometrical designs. (**B**) Confirmation of the experimental Poisson's ratios ($n = 10$) of plane substrate and the representative auxetic structures under tensile strains, ranging from 5×10^{-3} to 2×10^{-2}, fitted with the results of FEA. (**C** and **D**) GFs and resistive behaviors of the meta-crack sensors with various Poisson's ratio-modulated substrates (0.3 for plane substrate, −0.2 for rotachiral, −0.5 for vertical re-entrant, and −0.9 for horizontal re-entrant structures) through each overall strain sensing range with fitting lines. (**E**) Difference in strain sensing ranges for the various meta-crack sensors with diverse Poisson's ratios by different timing of complete electrical disconnection. (**F**) Resistance variation of the meta-crack sensor ($\nu = -0.9$) with 50 cycles of loading and unloading up to various peak strains (1×10^{-3}, 2×10^{-3}, and 2.9×10^{-3}). (**G**) Hysteresis behavior of the meta-crack sensor ($\nu = -0.9$) up to various peak strains (1×10^{-3}, 2×10^{-3}, and 2.9×10^{-3}) at a strain rate of 0.1 mm/min with a fitting line.

fitted using previously reported theoretical modeling for strain sensors based on induced-cracks (*33*). The theoretical modeling can elucidate the effect of Poisson's ratio using an opening rate parameter of the crack, providing an equation for the resistance calculation.

$$R = \left(\frac{1}{2} \left\{ 1 - \operatorname{erf}\left[\frac{\ln\left(\frac{k\varepsilon}{\delta_0}\right)}{\mu} \right] \right\} \right)^{-1} \quad (1)$$

The fitting parameters δ_0 and μ indicate the initial crack gap size and the deviation of the probability distribution, respectively, while ε represents the applied strain. The value k is a proportionality factor indicating the crack gap opening rate. The theoretical fitting shows k values of 13.54, 45.79, 97.66, and 140.14 for the Poisson's ratios of 0.3, −0.2, −0.5, and −0.9, respectively, presenting an inverse relation between the Poisson's ratio and k with a linear fitting (details in text S5 and table S2) (*31*).

There is a trade-off between strain resolution and maximum sensing limit, showing earlier electrical disconnection of the sensor with higher resolution in Fig. 2E. The maximum sensing strain, matched to the maximum measureable resistance depending on the range of the ohmmeter (here up to 2 megohms), can be defined as a critical strain shown as 2.9×10^{-3}, 1.59×10^{-2}, and 1.80×10^{-2} for Poisson's ratios of −0.9, −0.5, and −0.2, respectively. The lower Poisson's ratio leads to more rapid crack opening and earlier electrical disconnection, consequently resulting in a proportional relation between Poisson's ratio and the critical strain. Figure 2 (F and G) presents the raw resistance variations observed during cyclic loading-unloading of the sensor ($\nu = -0.9$), demonstrating its repeatability and hysteresis behavior up to a critical strain of 2.9×10^{-3} within the range where electrical disconnection does not occur. Cyclic tensile stretching comprising 50 cycles at each strain of 1×10^{-3}, 2×10^{-3}, and 2.9×10^{-3} (critical strain) with a sweeping speed of 0.3 mm/min shows repeated resistance variations while maintaining the same initial resistance level of 82.95 ± 1.21 kilohms (mean ± SD, further durability details in fig. S15), consistent with Fig. 2D. Figure 2G shows the hysteresis behavior of the sensor during loading and unloading at strain ranges from 0 to 1×10^{-3}, 2×10^{-3}, and 2.9×10^{-3} with a sweeping rate of 0.1 mm/min. The normalized resistance shows maximum variations of 12.56, 12.03, and 10.55% between the loading and unloading states at each strain range (different hysteresis behavior under various sweeping rates in fig. S16). The strain-normalized resistance behaviors align consistently with the fitting curve based on the theoretical modeling.

Parallel-deployed meta-cracks for overcoming trade-off between resolution and maximum sensing limit

The general challenge of a trade-off between strain resolution and maximum sensing limit of strain sensors can be overcome by designing a parallel circuit using a complementary combination of negative and positive Poisson's ratios. Figure 3A shows parallel-circuited sensor integrating components with both ratios in a parallel manner. Each component acts as an independent resistor changing resistance under uniformly applied strain (details including FEA results, in text S6 and fig. S17). Figure 3 (B and C) presents electrical network diagrams and working mechanisms of single-circuited and parallel-circuited sensors. The diagram describes the principle of extending the maximum strain sensing limit with hypersensitivity by using the advanced properties of each Poisson's ratio. The conductive layer with a single negative Poisson's ratio (Fig. 3B) and the negative Poisson's ratio portion of the parallel-circuited sensor (Fig. 3C) show electrical disconnection over critical strain. While the parallel-circuited sensor shows a negative Poisson's ratio-dependent high resistance change rate under critical strain, the electrical connection remains over the critical strain due to the positive Poisson's ratio. The high resistance change rate is maintained until the extended maximum limit of sensing strain. This combination integrates high sensitivity from extremely low strain and the extended measurable strain range for higher strain sensing.

The maximum sensing limit and sensitivity of parallel-circuited sensors can be adjusted by controlling the composition ratio and values of combined Poisson's ratios as shown in Fig. 3 (D and E). A parallel-circuited sensor with a composition ratio of 2:1 (ν −0.9:ν 0.3, symmetrically deployed in parallel) exhibited comparable maximum sensing strain (1.4×10^{-2}) and GF (6760 at 3×10^{-3} strain) to 0:1 and 1:0 single-circuited sensors, respectively, while maintaining GF values of more than 10,000 in a strain range from 4.0×10^{-3} to 1.4×10^{-2} (details of parallel-circuited sensors with different composition ratios and Poisson's ratios, and modified theoretical modeling for resistance variations in figs. S18 and S19, table S3, and text S7).

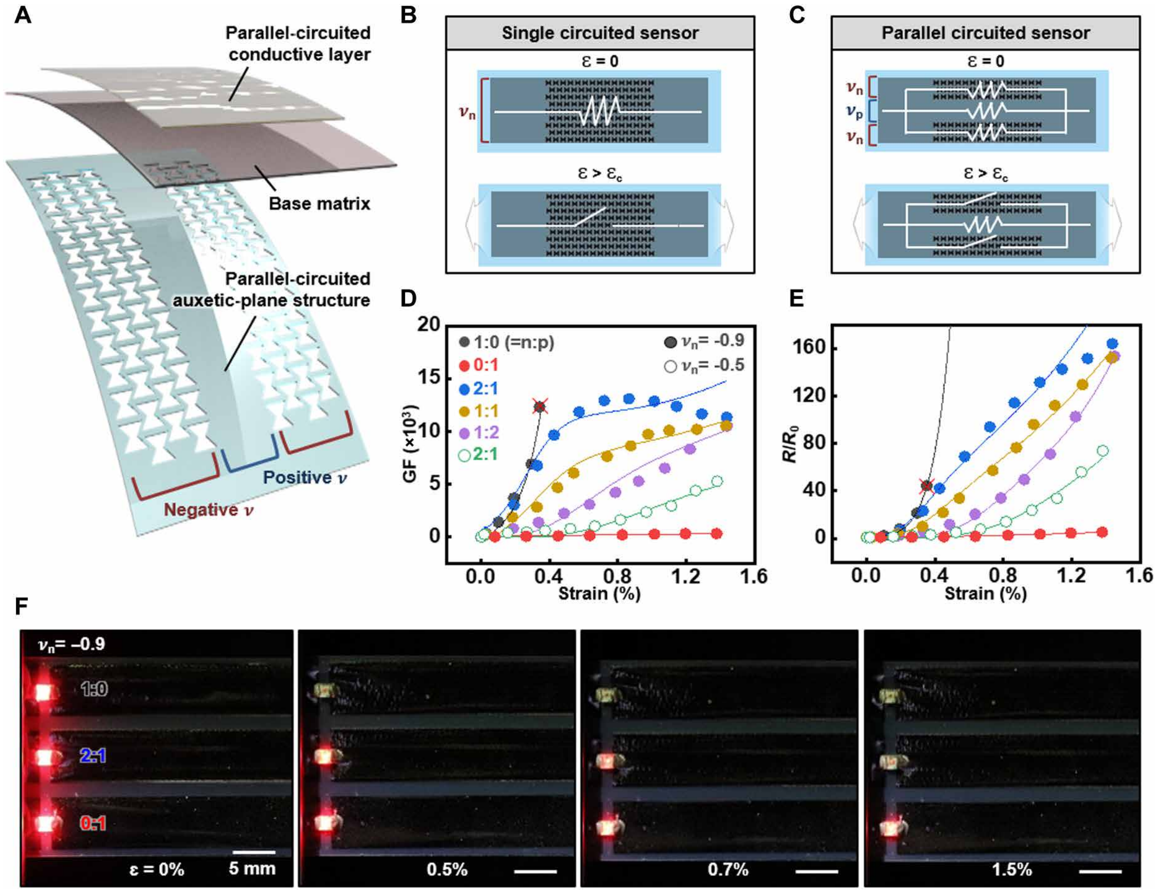

Fig. 3. Tuning of the sensing range of the meta-crack sensor by modifying the substrate design deploying parallel-circuited auxetic structures. (**A**) An exploded view illustration of the meta-crack sensor with a parallel-circuited auxetic-plane structure including both negative and positive Poisson's ratios. (**B** and **C**) Electrical disconnection behaviors of single-circuited sensor (only negative Poisson's ratio) and parallel-circuited sensor through the exertion of strain over critical strain in the form of electrical network diagram. Delay of complete electrical disconnection occurs in the parallel-circuited sensor. (**D** and **E**) GFs and resistive behaviors of the parallel-circuited meta-crack sensors with variously deployed plane and auxetic structures, including different composition ratios (2:1, 1:1, and 1:2) and negative Poisson's ratios (−0.5 and −0.9) through the extended strain sensing range with fitting lines. (**F**) Comparison of resistive behaviors under increasing applied strain between single-circuited sensors (only negative or positive Poisson's ratio) and parallel-circuited sensor (composition ratio of 2:1, negative Poisson's ratio of −0.9), estimated by the changes of LED light brightness. LED connected to the single-circuited sensors turns off immediately above the critical strain of 3×10^{-3} (top) or displays no change of brightness (bottom), while showing gradually diminishing brightness with the parallel-circuited sensor through whole strain sensing range (middle).

The theoretical fitting was well aligned, showing slight deviation due to interference of meta-substrate deformation at different locations (further discussion in text S7 and fig. S17).

Figure 3F and fig. S20 demonstrate the tunability of the maximum strain sensing limit and the strain resolution of the parallel-circuited sensor with a red light emitting diode (LED). Single-circuited sensors with negative and positive Poisson's ratios (−0.9 and 0.3) show an immediate light off at 5×10^{-3} strain (top) and no distinguishable brightness change up to 1.5×10^{-2} strain (bottom), while the parallel-circuited sensor with a composition ratio of 2:1 (ν −0.9:ν 0.3) shows a gradually diminishing brightness under rising strain in the overall sensing range of 0 to 1.5×10^{-2} strain (middle).

MEMS application with a structural adaptability

The thin film–based soft and flexible meta-crack sensor offers structural adaptability in microelectromechanical system (MEMS) applications, including hypersensitive monitoring of strain, pressure, and force. Figure 4A shows an illustration and photograph of a representative MEMS application using a trench structure (trench size: 3 mm by 3 mm by 1 mm) and the meta-crack sensor (cracked metal film dimension: 2.5 mm by 0.25 mm by 50 nm). The meta-crack sensor, attached right above the middle edge of the PDMS trench, experiences maximum concentrated strains due to mechanical deflection induced by temperature variation or physical touch. Figure 4 (B and C) and fig. S21 demonstrate the monitoring of minute pressure-induced strains generated by air expansion during temperature variation. The deflection of the flexible substrate of the sensor is induced by a heat source right below the trench. The thermal expansion occurs both on the sensor and on the trapped air in the trench, consequently inducing strain on the sensor by the expansion itself and the air-generated outward pressure, confirmed by FEA. Figure 4C shows the minute strain variation (up to 7×10^{-4} strain) with the

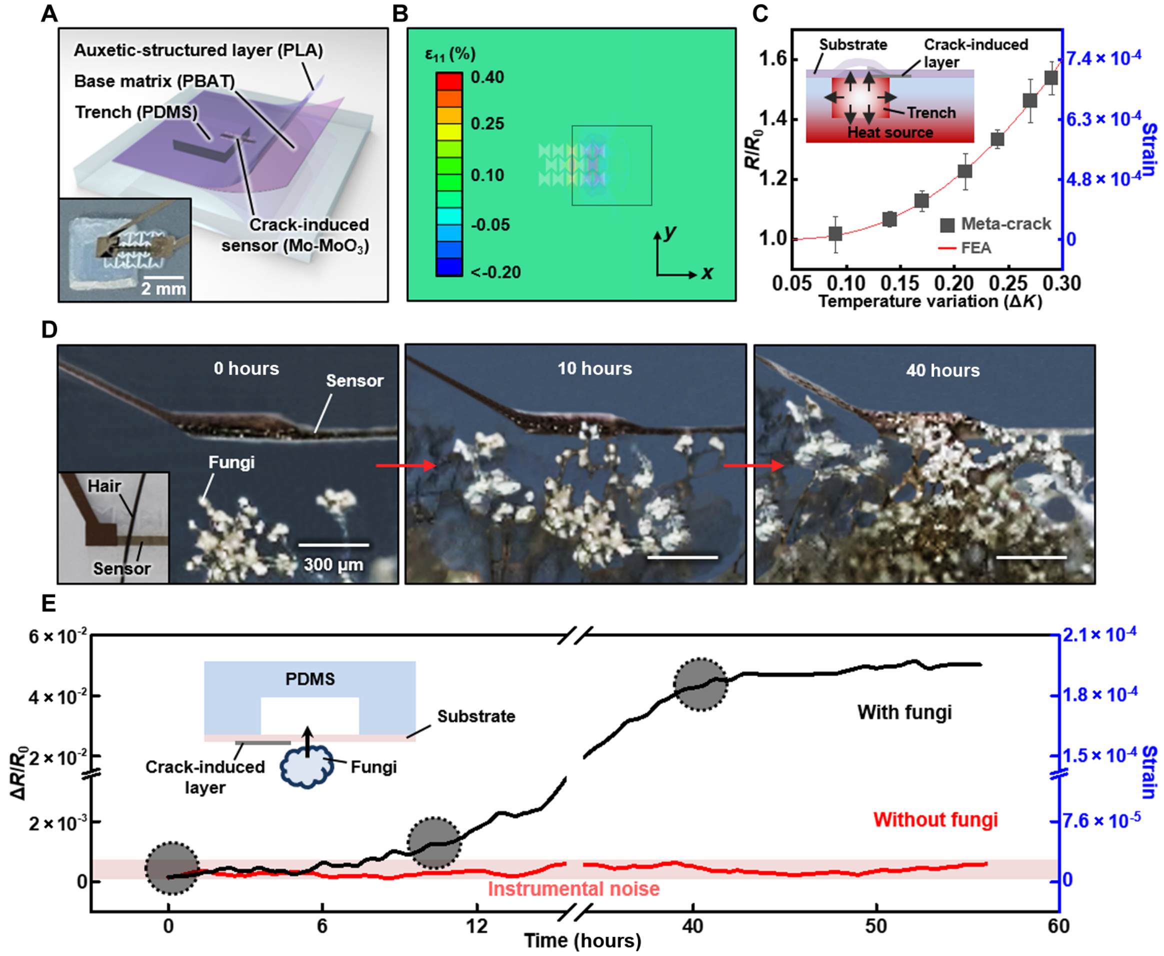

Fig. 4. Structural adaptability of the meta-crack sensor integrated with MEMS for various monitoring applications. (**A**) Illustration and a photograph (inset) of the meta-crack sensor on a PDMS trench. (**B**) Confirmation of induced strain distribution on the meta-crack sensor integrated with the PDMS trench by beneath heat source based on FEA. (**C**) Temperature sensitivity of the trench-integrated meta-crack sensor within 0.3 K variation, fitted to converted normalized resistance based on strains derived from FEA and a calibration curve obtained by the sensor. A schematic illustration (inset) shows a cross-sectional view of the meta-crack sensor on trench with a heat source right below the trench. Combined effect of thermal expansion of the sensor and generated outward pressure by thermal expansion of the trapped air in the trench leads to deflection of the flexible substrate, inducing applied strain on the sensor. (**D** and **E**) Demonstration of the trench-integrated meta-crack sensor for real-time monitoring of growing fungi, with step-by-step photographs displaying the approaching fungi toward the above fixed sensor (0 hours), initial touch between the fungi and the sensor (10 hours), and complete contact between a bundle of fungi and the sensor (40 hours). The initial touch of the growing fungi induces normalized resistance variation over instrumental noise, indicating an applied strain of 10^{-5} scale. Inset photograph in (D) shows a relative dimension of the sensor compared to a hair.

trench-integrated meta-crack sensor exhibiting distinguishable normalized resistance under temperature variation within a range of 0.3 K (no notable effect of the temperature variation on metal film resistance with a small thermal coefficient of resistance, 3.66×10^{-4}, in fig. S4). The FEA-based fitting line is obtained by converting simulated strains to normalized resistance with the aid of a strain calibration curve.

Figure 4 (D and E) and fig. S22 demonstrate the detection of extremely small strain using the MEMS structure, continuously monitoring the growing force of fungi (*Rhizopus stolonifer* species). The meta-crack sensor with MEMS detected the upward-growing fungi initially softly touching the freestanding substrate of the sensor (inset of Fig. 4E, fixed at the top), showing notable normalized resistance changes initially, indicating an induced strain of 6.3×10^{-5}. The estimated touching force and vertical displacement of the deflection are about 3 μN and 2 μm, respectively, based on FEA results considering the fungi-induced strain (details in text S8 and fig. S23). A bunch of fungi contacted the substrate after 40 hours and stopped the marked growth, inducing a somewhat saturated strain of 1.9×10^{-4} measured by the meta-crack sensor.

In vivo real-time, wireless monitoring of cerebrovascular dynamics with conformal coverages

The meta-crack sensor enables real-time continuous, shape-adaptable monitoring and spatial mapping with conformal coverage on universal targets based on readily fabricated array-type thin film forms. The flexible and soft sensing platform, with its hypersensitivity, is suitable for biological systems and is especially applicable to real-time monitoring of cerebrovascular dynamics in locations distant from the heart. It allows for the early detection of abnormal intracranial pressure variation during traumatic brain injury and the exploration of unknown mechanobiology, which was previously impossible due to low sensitivity. Figure 5 demonstrates the real-time monitoring of minute biomechanical signals related to cerebrovascular dynamics on the surface of the cerebral cortex in a canine model, previously considered challenging due to infinitesimal strain variation up to the 10^{-4} level (*26*). Figure 5A shows an illustration of an overall meta-mesh crack sensor array mapping the distribution of blood pressure changes of adjacent cerebral vessels by monitoring their gradually varying surface strains up to the 10^{-4} level with 10^{-5} scale resolution for accurate feature detection (details of the optimized mesh-structured substrate for conformal coverage and as-is deformation in text S9 and figs. S24 to S26) (*37–39*).

Figure 5B and fig. S27 show photographs and a schematic illustration of the monitoring setup for the mechanical signal of the brain in the canine model. The meta-mesh crack sensor array is attached to the dura mater of the brain in a skull-removed state, interfaced with wires electrically connecting the seven sensors and a multichannel digital multimeter. The surface strain of the dura mater periodically changes due to the underlying cerebral vessels transferring the blood pressure variation.

Figure 5 (C and D), texts S10 and 11, and figs. S28 to S30 summarize the results of real-time pulse pressure and rate, and blood flow monitoring on the cerebral vessels (fabrication procedures and photographs of sensors in figs. S31 and S32). Figure 5C shows specific pulsatile features and the distribution of averaged pulse pressure expressed as colorized normalized resistance. The meta-mesh crack sensor detected certain vascular pressure patterns related to cardiovascular health, including a peak systolic pressure caused by maximum left ventricular contraction, a dicrotic notch due to aortic valve closure, and the following small peak by tricuspid valve opening (*40*). Meanwhile, the plane crack sensor (GF 10 at 10^{-4} strain in Fig. 2C) only provided the peak systolic pressure pattern with normalized resistance of smaller absolute values (less than a quarter) due to low sensitivity. Figure 5D shows blood flow aspects through a bundle of vessels within 0.75 s with the meta-mesh crack sensor array by analyzing the real-time mapping of the overall surface strain distribution. The strain on the vessel increases immediately where the blood flows, and then decreases right after passing through. The time frame mapping indicates the direction and rate of the blood flow, implying the vascular health state related to vasoocclusion (*7*).

Figure 5 (E to G) and figs. S33 to S37 show the integration of an NFC-based system-on-chip with the meta-crack sensor for in vivo demonstration of on-board hypersensitive, wireless strain monitoring (details for fabrication of the NFC-based sensing module in text S12). The wireless sensing module attached to the dura mater above the vessels of the canine brain model enabled cerebrovascular pulse pressure and rate monitoring involving the administration of epinephrine. Figure 5 (F and G) shows the normal blood-flowing state initially and the excited state following epinephrine injection, which induced contraction of vascular smooth muscle, tightening the blood vessels (*41*). The excited state lasted over 2 min, including 2.7 times increased peak systolic pressure, a proportionally deepened dicrotic notch, and 1.5 times the pulse rate with a gradual increase in the pressure baseline. The state then returned to normal, showing a stable pulse condition (details in figs. S36 and S37) (*35*, *42*, *43*).

Overall materials composing the meta-crack sensor show biodegradability, presenting the potential for the various in vivo applications requiring short-term biomechanical signal monitoring, such as traumatic brain injuries where intracranial pressure increases within days (*20*, *35*). In addition, the fully biodegradable nature of the sensor prevents issues of permanent implantation and secondary surgeries incurring device migration, abnormal organ growth, tissue damage, and infection risks (*20*). Ultimately, the hypersensitive and fully biodegradable meta-crack sensor is available for these applications with specific advantages. The encapsulation of the sensor is essential for stable operation and data reliability in biofluidic conditions due to its biodegradability. The performance of the encapsulation materials for blocking the penetration of water molecules depends on the hydrophobicity and thickness of the materials (*35*). Meanwhile, a trade-off between the GF and thickness of the encapsulation materials results in a disadvantage for strain sensitivity in exchange for an enhanced lifetime of the sensor (*35*). Here, a 150-μm-thick biodegradable encapsulation membrane composed of a candelilla wax-bees wax-PBAT blend was used in consideration of the compromised sensitivity (decreased GF to 22.2%) and lifetime (up to 3 days) (*35*). The encapsulation provided operation stability and reliability for 3 days in both in vitro [1 M phosphate-buffered saline (PBS), pH 7.4, 18°C] and in vivo (surface of the cerebral cortex in a rat brain model) environments (fig. S38). A stable base resistance over 3 days showed normalized resistance noise (SDs) of 0.0001 and 0.0011, respectively, due to environmental and respiratory noise. These variations, same with signals induced by the 10^{-5} scale strain, were much lower than the resistance changes induced by strains on the target blood vessel (10^{-4} scale strain, fig. S28).

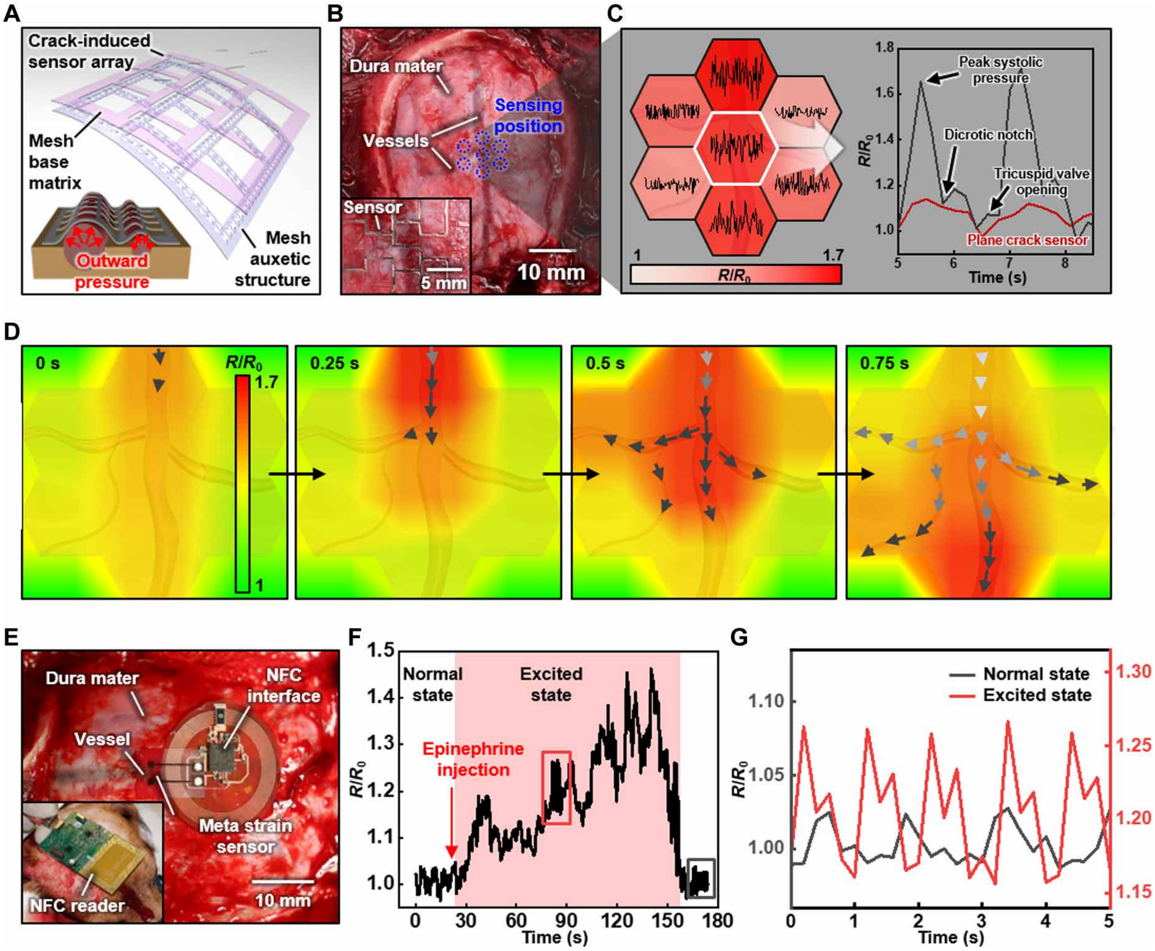

Fig. 5. In vivo monitoring of cerebrovascular dynamics on the canine brain and the integration of the NFC system for wireless monitoring. (**A**) Illustration of the vessel-embedded skin and the mesh type meta-crack sensor array on the skin surface. The mesh type sensor deforms well in high compliance with the application of an outward pressure on the vessels. (**B**) Enlarged photographs of dura mater surface of the canine brain including several vessels, and the attached mesh type meta-crack sensor array consists of seven sensors (inset). (**C**) Vessel pressure-induced surface strain distribution acquired from each sensor in normalized resistance with the color map, where the color indicates the averaged amplitude of the normalized resistance changes for 10 s. The acquired signals in each position show temporal characteristics of the surface strain change for a duration over 10 s. Distinguishable features of the vascular pressure variation, including peak systolic pressure, dicrotic notch, and tricuspid valve opening-induced following small peak, observed exceptionally using the meta-mesh crack sensors compared to the monitoring by plane crack sensors. (**D**) Spatiotemporal mapping of cerebrovascular blood flow from the meta-mesh crack sensor array for a second with a time interval of 0.25 s. (**E**) Photographs of the meta-crack sensor-integrated NFC module on the dura mater of the canine brain for monitoring of cerebrovascular pressure variation, and demonstration of wireless sensing (inset). (**F**) Real-time wireless monitoring of the vascular pressure-induced surface strain signals in normalized resistance variation during epinephrine injection inducing an excited state with an increase of the vascular pressure. (**G**) Enlarged normalized resistance variation for 5 s in the normal and excited states, showing 2.7 and 1.5 times increased peak systolic pressure and pulse rate, respectively, in the excited state.

DISCUSSION

In summary, hypersensitive meta-crack sensors within infinitesimal strains offer real-time continuous and shape-adaptable monitoring of minute mechanical deformations in a truly ultrasensitive manner. While the sensors are implemented using a few representative negative Poisson's ratios, diverse negative Poisson's ratios can be realized by using a wider range of auxetic structure designs and optimizing the filling ratio of auxetic patterns, allowing for the modeling and experimental validation of further previously undiscovered crack-opening behaviors. This would enable targeted amplification and filtering of resistance signals in specific strain regions, leading to a more optimized strain sensitivity for strategic applications. The meta-crack sensors exhibit versatile characteristics, including an exceptional detection limit for strain and force, a tunable sensing range overcoming the trade-off with sensing resolution, integration capability with MEMS structures for various applications, and feasibility as promising tools for monitoring cerebrovascular dynamics with a post–signal processing-free system. In addition, integrating a propagation stopping layer for crack depth control in crack-based sensors could enable ultrastable and tough properties, making them suitable for applications requiring durability over hundreds of thousands of cycles (*44*). These free-form sensors, acting as mechanoelectric indicators, have the potential to constitute unprecedented functional platforms for monitoring brain activities accompanied by localized swelling with brain-wrapping forms, monitoring three-dimensional (3D) tactile cellular responses including focal adhesion-inducing

cytoskeletal dynamics, and quality control of differentiated or chemically treated cells. These promising approaches would make remarkable contributions to advanced early diagnosis and efficient prognosis assessment for brain diseases, as well as tools for comprehensive understanding of cellular functionalities during homeostasis, wound healing, and therapeutic processes.

MATERIALS AND METHODS

Fabrication and characterization of meta-crack sensor and conventional crack sensor

A thin film of PBAT (30 μm, S-EnPol, Korea) was prepared by spin coating of PBAT solution (0.3 g/ml) on the glass substrate. Thin film adhesive layer (MoO_3, 15 nm) and metal layer (Mo, 50 nm) were sequentially directly deposited on the PBAT film by a sputtering system (J Vacuum Technology, Korea). The film was peeled off from glass substrate, and cyclic tensile strain (~2%, 20 cycles) was applied to form the crack structure on the metal film. A thin film of PLA (10 μm, Sindoh, Korea) was prepared by spin coating of PLA solution (0.3 g/ml) on the glass substrate. The PLA substrate was patterned with an auxetic structure (Poisson's ratio, −0.9) using a UV laser (MD-U1000C, Keyence, Japan). The two thin film substrates were bonded through a thermal bonding process. The conventional crack sensor was fabricated with the Mo/MoO_3 (50 nm/15 nm) film deposited (sputtering process, J Vacuum Technology, Korea) on no patterned plane PBAT/PLA (30 μm/10 μm) substrate, followed by exertion of cyclic tensile strain (~2%, 20 cycles) to form the uniform crack structures. The resistance changes during an applied tensile strain of 3×10^{-3} and 4.5×10^{-4} were measured by a digital multimeter (PXIe-4081, National Instruments, USA). The meta-crack sensors with different auxetic structures (Poisson's ratios of −0.5 and −0.2) were prepared with the UV laser (MD-U1000C, Keyence, Japan) patterning on the PLA substrate.

In situ observation of crack opening behavior using an SEM

The crack structure of the meta-crack sensor and conventional crack sensor was observed by an SEM (ZEISS GeminiSEM 560, Germany). The open state (applied strain of 0.5% by a custom tensile stretching jig) of the crack in each sensor at the same location was observed by an SEM (ZEISS GeminiSEM 560, Germany).

Simulation of crack widening behavior with diverse crack morphologies and Poisson's ratios

FEA was conducted using the commercially available software package, ABAQUS. Models for analysis of the crack gap opening behavior in a film-on-substrate crack sensor were designed, focusing on the effect of the substrate's Poisson's ratio. A metal film with a thickness of 50 nm was applied to a substrate with a thickness of 2 μm. The metal film layer was shaped into a sine curve to form angles (θ) between the tensile-perpendicular direction and the crack edge, varying from 10° to 80° with an assumption that the crack penetrated through the full thickness of the metal layer. Displacement between two parallel crack edges was measured at the top surface of the metal layer. The surfaces perpendicular to the stretching direction were constrained with a displacement boundary condition to stretch the sample up to a total strain of 2%. Lateral displacements of the side surfaces were set to apply periodic boundary conditions. The mechanical properties of the materials were assumed to follow linear elastic behavior, with the metal having an elastic modulus of 300 GPa and a Poisson's ratio of 0.3, and the substrate having an elastic modulus of 2.3 GPa. The Poisson's ratio of the substrate was varied among −0.9, −0.5, −0.2, and 0.3. The model used C3D8 linear elements.

Measurement of the Poisson's ratio of plane substrate and diverse meta-substrates

The plane substrate (Poisson's ratio of 0.3) and meta-substrates consisting of diverse auxetic structures (Poisson's ratios of −0.9, −0.5 and −0.2) were prepared with the UV laser patterning (for meta-substrates, MD-U1000C, Keyence, Japan) on the PLA substrate. The substrates were stretched by a custom tensile stretching jig up to 2% strain with 0.5% interval. In each applied strain, the fixed four points (two points in a vertical line, two points in a horizontal line) were imaged, and the x- and y-axis displacements measured by the ImageJ software were used to calculate the Poisson's ratios in 10 samples ($n = 10$).

FEA for comparison of the Poisson's ratio of plane substrate and diverse meta-substrates

FEA was conducted using the commercially available standard software package, ABAQUS. Models for analysis of the effect of various auxetic structures of the PLA layer on the surface strain distribution of PBAT were designed as in fig. S7 (unit geometries of the auxetic structures used in Fig. 2). Line width of the structures repeating the unit geometries was fixed as 100 μm. PLA substrates with the repeated unit geometries were modeled with a thickness of 10 μm, the same dimension used for the experiment. The PBAT-PLA multilayer system was designed with thicknesses of 30 and 10 μm, respectively, with the interface constrained using the tie option. The four side surfaces of the PLA layer were constrained to four different reference points using the coupling option. The multilayer materials were stretched in the axial direction using two of these reference points, which were set with displacement boundary conditions. The Poisson's ratio of the different auxetic structures was calculated based on the displacement of these reference points. The mechanical properties of the materials were assumed to follow linear elastic behavior, with PBAT having an elastic modulus of 23.5 MPa and a Poisson's ratio of 0.4, and PLA having an elastic modulus of 3.5 GPa and a Poisson's ratio of 0.32. The model used C3D8 linear elements.

Durability of the meta-crack sensor

The meta-crack sensor with a Poisson's ratio of −0.9 was tested for long cyclic tensile test (extended cycle number of 10,000 and 4000 for the 0.1% and 0.29% strain, respectively) to confirm durability of the sensor as shown in fig. S15. The cyclic tensile test was conducted with a universal testing system (Instron, USA) and the resistance of the sensor was recorded continuously with a digital multimeter (PXIe-4081, National Instruments, USA). The tensile stretching sweeping rate was fixed to 10 mm/min for both the 0.1% and 0.29% strain. The peak normalized resistance remains at the same level of 1.46 ± 0.006 and 19.07 ± 0.149 (mean ± SD) until 6000 and 2000 cycles for the 0.1% and 0.29% strain. The sensor exhibited a stable range up to 6400 cycles while inducing 0.1% cyclic strain and up to 2200 cycles while inducing 0.29% strain.

Fabrication of a pressure sensor for monitoring of minute mechanical signals

The trench-type pressure sensing device was fabricated for monitoring of minute mechanical signals including an infinitesimal

pressure difference-induced strain by temperature variation and fungi touching-induced force and corresponding strain as shown in Fig. 4A. The PDMS trench with dimension of 3 mm by 3 mm by 1 mm (width, length, and depth) was fabricated using a PLA negative mold printed with a 3D printer (Sindoh 3DWOX, Korea). The crack-induced sensor with dimensions of 2.5 mm by 0.25 mm (width and length) was placed on the middle of the edge of the trench to maximize the strain induced by the deflection of the substrate. The deflection of the substrate deformed the sensor, inducing tensile strain on the conductive layer (Mo/MoO_3), as shown in figs. S21 and S23.

Simulation of heat source- and fungi-induced pressure sensing with mechanical deformation

FEA was conducted using the commercially available standard software package, ABAQUS. Models were designed for analysis of strain and displacement level, when trench was deformed by heat source– and fungi-induced pressure. The PDMS trench was covered with a double-layer substrate of PBAT-PLA, with a thickness of 30 and 10 μm (Fig. 4A), respectively, with the interface constraint using tie option. Specifically for the fungi-induced pressure sensing, geometry of the PDMS trench and meta-crack sensor was modeled based on the experiment in Fig. 4 (D and E), and the force was applied on the middle region of the trench assuming that the fungi apply a point load on the middle of the PBAT base matrix. Surface pressure and concentrated force were applied to simulate an air pressure increase of 371 Pa by heat source and a contact force of 3 μN by fungi. Inner pressure increase was calculated by ideal gas expansion by a temperature increase of 1°C in Fig. 4 (B and C).

In vivo demonstration for biomechanical signal monitoring using rat and canine brain models

All animal experiments were conducted following both national and institutional guidelines and the Guide for the Care and Use Committees of laboratory animals based on protocols approved by the ASAN Institute for Life Sciences at the Asan Medical Center (2022-14-012) and the KBIO Osong Medical Innovation Foundation (KBIO-IACUC-2021-272 and KBIO-IACUC-2022-103). Rat models (Sprague-Dawley rat, 14 weeks, male, 400 to 420 g) and canine models (Beagle, 8 to 10 months, male, 10 to 13 kg) were used for in vivo verification of biomechanical signal monitoring on the brain. The animal models were anesthetized with Zoletil [5 mg/kg, intramuscularly (IM)] and xylazine (2 mg/kg, IM) and maintained under the state of anaesthesia with isoflurane (<3%). The skin and subcutaneous layers of the surgical site were incised with scissors. The craniotomy was done with a surgical drill (REF 7020-001, CONMED) to make a skull hole (hole diameter, ~10 mm for rat and ~50 mm for the canine model) for brain demonstration. The surgical locations were sutured using polyglycolic acid suture. The state of elevated blood pressure was induced by an administration of epinephrine (10 μg/kg, intravenously).

Fabrication process of various types of array sensors for in vivo demonstration

Four types of substrates (plane, auxetic structured, mesh structured, auxetic and mesh structured) were fabricated to monitor real-time pulse pressure and rate on cerebral vessel bundles. A total of five types of sensors were fabricated, including sensors integrating Mo crack-induced layers on each substrate and sensors integrating Ag nanowires on auxetic– and mesh–structured substrates. The plane substrate was prepared by spin coating of PBAT solution (0.3 g/ml, Sigma-Aldrich, USA) onto a glass substrate to create a PBAT film (~30 μm) and spin coating of PLA solution (0.3 g/ml, Sigma-Aldrich, USA) to create a PLA film (~10 μm). The PLA film was adhered to the PBAT film through a thermal bonding process. Subsequently, a Mo/MoO_3 film (50 nm/15 nm) was deposited directly onto the PBAT film using a sputtering system (J Vacuum Technology, Korea), then peeled off from the glass substrate and mounted on a custom tensile stretching jig to induce cracks. Cyclic tensile strain (~2%, 20 cycles) was applied, followed by sputtering of Cu lines (100 nm) to fabricate the sensor interface (fig. S31A). For the auxetic– and mesh–structured substrate, PBAT and PLA films were prepared similarly. The PLA substrate was patterned with an auxetic structure (Poisson's ratio, −0.9) using a UV laser (MD-U1000C, Keyence, Japan), while the PBAT substrate had a Mo/MoO_3 film (50 nm/15 nm) deposited directly. The substrates were then subjected to cyclic tensile strain (~2%, 20 cycles) to induce cracks. The two substrates were bonded through a thermal bonding process, and the double-layer substrate was patterned into a mesh design optimized in fig. S26 using the UV laser. Cu lines (100 nm) were then sputtered to create the sensor interface (fig. S31B). For the auxetic-structured substrate, PBAT and PLA films were prepared in the same manner. The PLA substrate was patterned with an auxetic structure (Poisson's ratio, −0.9) using the UV laser, and the PBAT substrate had a Mo/MoO_3 film (50 nm/15 nm) deposited. Cyclic tensile strain (~2%, 20 cycles) was applied to induce cracks. The substrates were bonded through a thermal bonding process, followed by sputtering of Cu lines (100 nm) to fabricate the sensor interface (fig. S31C). For the mesh-structured substrate, PBAT and PLA films were prepared as described before. The PBAT substrate had a Mo/MoO_3 film (50 nm/15 nm) deposited, and cyclic tensile strain (~2%, 20 cycles) was applied to induce cracks. The substrates were bonded through a thermal bonding process, and the double-layer substrate was patterned into a mesh design using the UV laser, as optimized in fig. S26. Cu lines (100 nm) were then sputtered to create the sensor interface (fig. S31D). In the fabrication of the Ag nanowire sensor on the auxetic– and mesh–structured substrate, the basic substrate fabrication process was identical to that of the auxetic– and mesh–structured crack sensor. However, the top conductive layer was replaced with an Ag nanowire sheet instead of the crack-induced Mo layer. The Ag nanowire sheet was prepared by spraying 0.3 ml of AgNWs ethanol solution (4 mg/ml, Sigma-Aldrich, USA).

Supplementary Materials

This PDF file includes:
Supplementary Text S1 to S12
Figs. S1 to S38
Tables S1 to S3
References

REFERENCES AND NOTES

1. A. Mongera, M. Pochitaloff, H. J. Gustafson, G. A. Stooke-Vaughan, P. Rowghanian, S. Kim, O. Campàs, Mechanics of the cellular microenvironment as probed by cells in vivo during zebrafish presomitic mesoderm differentiation. *Nat. Mater.* **22**, 135–143 (2023).
2. D. J. Shiwarski, J. W. Tashman, A. Tsamis, J. M. Bliley, M. A. Blundon, E. Aranda-Michel, Q. Jallerat, J. M. Szymanski, B. M. McCartney, A. W. Feinberg, Fibronectin-based nanomechanical biosensors to map 3D surface strains in live cells and tissue. *Nat. Commun.* **11**, 5883 (2020).
3. B. E. Danielsson, B. G. Abraham, E. Mäntylä, J. I. Cabe, C. R. Mayer, A. Rekonen, F. Ek, D. E. Conway, T. O. Ihalainen, Nuclear lamina strain states revealed by intermolecular force biosensor. *Nat. Commun.* **14**, 3867 (2023).

4. M. Chatterjee, S. Özdemir, C. Fritz, W. Möbius, L. Kleineidam, E. Mandelkow, J. Biernat, C. Doğdu, O. Peters, N. C. Cosma, X. Wang, L.-S. Schneider, J. Priller, E. Spruth, A. A. Kühn, P. Krause, T. Klockgether, I. R. Vogt, O. Kimmich, A. Spottke, D. C. Hoffmann, K. Fliessbach, C. Miklitz, C. M. Cormick, P. Weydt, B. Falkenburger, M. Brandt, R. Guenther, E. Dinter, J. Wiltfang, N. Hansen, M. Bähr, I. Zerr, A. Flöel, P. J. Nestor, E. Düzel, W. Glanz, E. Incesoy, K. Bürger, D. Janowitz, R. Perneczky, B. S. Rauchmann, F. Hopfner, O. Wagemann, J. Levin, S. Teipel, I. Kilimann, D. Goerss, J. Prudlo, T. Gasser, K. Brockmann, D. Mengel, M. Zimmermann, M. Synofzik, C. Wilke, J. Selma-González, J. Turon-Sans, M. A. Santos-Santos, D. Alcolea, S. Rubio-Guerra, J. Fortea, Á. Carbayo, A. Lleó, R. Rojas-García, I. Illán-Gala, M. Wagner, I. Frommann, S. Roeske, L. Bertram, M. T. Heneka, F. Brosseron, A. Ramirez, M. Schmid, R. Beschorner, A. Halle, J. Herms, M. Neumann, N. R. Barthélemy, R. J. Bateman, P. Rizzu, P. Heutink, O. Dols-Icardo, G. Höglinger, A. Hermann, A. Schneider, Plasma extracellular vesicle tau and TDP-43 as diagnostic biomarkers in FTD and ALS. *Nat. Med.* **30**, 1771–1783 (2024).
5. L. Wang, X. Wang, Y. Wu, M. Guo, C. Gu, C. Dai, D. Kong, Y. Wang, C. Zhang, D. Qu, C. Fan, Y. Xie, Z. Zhu, Y. Liu, D. Wei, Rapid and ultrasensitive electromechanical detection of ions, biomolecules and SARS-CoV-2 RNA in unamplified samples. *Nat. Biomed. Eng.* **6**, 276–285 (2022).
6. C. Pang, G.-Y. Lee, T.-I. Kim, S. M. Kim, H. N. Kim, S.-H. Ahn, K.-Y. Suh, A flexible and highly sensitive strain-gauge sensor using reversible interlocking of nanofibres. *Nat. Mater.* **11**, 795–801 (2012).
7. C. M. Boutry, L. Beker, Y. Kaizawa, C. Vassos, H. Tran, A. C. Hinckley, R. Pfattner, S. Niu, J. Li, J. Claverie, Z. Wang, J. Chang, P. M. Fox, Z. Bao, Biodegradable and flexible arterial-pulse sensor for the wireless monitoring of blood flow. *Nat. Biomed. Eng.* **3**, 47–57 (2019).
8. C. M. Boutry, Y. Kaizawa, B. C. Schroeder, A. Chortos, A. Legrand, Z. Wang, J. Chang, P. Fox, Z. Bao, A stretchable and biodegradable strain and pressure sensor for orthopaedic application. *Nat. Electron.* **1**, 314–321 (2018).
9. O. A. Araromi, M. A. Graule, K. L. Dorsey, S. Castellanos, J. R. Foster, W.-H. Hsu, A. E. Passy, J. J. Vlassak, J. C. Weaver, C. J. Walsh, R. J. Wood, Ultra-sensitive and resilient compliant strain gauges for soft machines. *Nature* **587**, 219–224 (2020).
10. S. Liu, J. Zhang, Y. Zhang, R. Zhu, A wearable motion capture device able to detect dynamic motion of human limbs. *Nat. Commun.* **11**, 5615 (2020).
11. G. Valle, N. Katic Secerovic, D. Eggemann, O. Gorskii, N. Pavlova, F. M. Petrini, P. Cvancara, T. Stieglitz, P. Musienko, M. Bumbasirevic, S. Raspopovic, Biomimetic computer-to-brain communication enhancing naturalistic touch sensations via peripheral nerve stimulation. *Nat. Commun.* **15**, 1151 (2024).
12. M. C. Benn, S. A. Pot, J. Moeller, T. Yamashita, C. M. Fonta, G. Orend, P. Kollmannsberger, V. Vogel, How the mechanobiology orchestrates the iterative and reciprocal ECM-cell cross-talk that drives microtissue growth. *Sci. Adv.* **9**, eadd9275 (2023).
13. N. Marina, I. N. Christie, A. Korsak, M. Doronin, A. Brazhe, P. S. Hosford, J. A. Wells, S. Sheikhbahaei, I. Humoud, J. F. R. Paton, M. F. Lythgoe, A. Semyanov, S. Kasparov, A. V. Gourine, Astrocytes monitor cerebral perfusion and control systemic circulation to maintain brain blood flow. *Nat. Commun.* **11**, 131 (2020).
14. A. Senoussi, J.-C. Galas, A. Estevez-Torres, Programmed mechano-chemical coupling in reaction-diffusion active matter. *Sci. Adv.* **7**, eabi9865 (2021).
15. F. J. Armistead, J. Gala De Pablo, H. Gadêlha, S. A. Peyman, S. D. Evans, Physical biomarkers of disease progression: On-chip monitoring of changes in mechanobiology of colorectal cancer cells. *Sci. Rep.* **10**, 3254 (2020).
16. A. Abramson, C. T. Chan, Y. Khan, A. Mermin-Bunnell, N. Matsuhisa, R. Fong, R. Shad, W. Hiesinger, P. Mallick, S. S. Gambhir, Z. Bao, A flexible electronic strain sensor for the real-time monitoring of tumor regression. *Sci. Adv.* **8**, eabn6550 (2022).
17. M. Plodinec, M. Loparic, C. A. Monnier, E. C. Obermann, R. Zanetti-Dallenbach, P. Oertle, J. T. Hyotyla, U. Aebi, M. Bentires-Alj, R. Y. H. Lim, C.-A. Schoenenberger, The nanomechanical signature of breast cancer. *Nat. Nanotechnol.* **7**, 757–765 (2012).
18. J.-Y. Yoo, S. Oh, W. Shalish, W.-Y. Maeng, E. Cerier, E. Jeanne, M.-K. Chung, S. Lv, Y. Wu, S. Yoo, A. Tzavelis, J. Trueb, M. Park, H. Jeong, E. Okunzuwa, S. Smilkova, G. Kim, J. Kim, G. Chung, Y. Park, A. Banks, S. Xu, G. M. Sant'Anna, D. E. Weese-Mayer, A. Bharat, J. A. Rogers, Wireless broadband acousto-mechanical sensing system for continuous physiological monitoring. *Nat. Med.* **29**, 3137–3148 (2023).
19. Y. J. Kang, H. M. Arafa, J.-Y. Yoo, C. Kantarcigil, J.-T. Kim, H. Jeong, S. Yoo, S. Oh, J. Kim, C. Wu, A. Tzavelis, Y. Wu, K. Kwon, J. Winograd, S. Xu, B. Martin-Harris, J. A. Rogers, Soft skin-interfaced mechano-acoustic sensors for real-time monitoring and patient feedback on respiratory and swallowing biomechanics. *npj Digit. Med.* **5**, 147 (2022).
20. S.-K. Kang, R. K. J. Murphy, S.-W. Hwang, S. M. Lee, D. V. Harburg, N. A. Krueger, J. Shin, P. Gamble, H. Cheng, S. Yu, Z. Liu, J. G. McCall, M. Stephen, H. Ying, J. Kim, G. Park, R. C. Webb, C. H. Lee, S. Chung, D. S. Wie, A. D. Gujar, B. Vemulapalli, A. H. Kim, K.-M. Lee, J. Cheng, Y. Huang, S. H. Lee, P. V. Braun, W. Z. Ray, J. A. Rogers, Bioresorbable silicon electronic sensors for the brain. *Nature* **530**, 71–76 (2016).
21. T. Kim, Y. Shin, K. Kang, K. Kim, G. Kim, Y. Byeon, H. Kim, Y. Gao, J. R. Lee, G. Son, T. Kim, Y. Jun, J. Kim, J. Lee, S. Um, Y. Kwon, B. G. Son, M. Cho, M. Sang, J. Shin, K. Kim, J. Suh, H. Choi, S. Hong, H. Cheng, H.-G. Kang, D. Hwang, K. J. Yu, Ultrathin crystalline-silicon-based strain gauges with deep learning algorithms for silent speech interfaces. *Nat. Commun.* **13**, 5815 (2022).
22. J. Guo, B. Zhou, R. Zong, L. Pan, X. Li, X. Yu, C. Yang, L. Kong, Q. Dai, Stretchable and highly sensitive optical strain sensors for human-activity monitoring and healthcare. *ACS Appl. Mater. Interfaces* **11**, 33589–33598 (2019).
23. J. Guo, M. Niu, C. Yang, Highly flexible and stretchable optical strain sensing for human motion detection. *Optica* **4**, 1285–1288 (2017).
24. X. Guo, W. Hong, T. Zhang, H. Li, T. Zhu, Q. Hong, D. Wang, L. Liu, Y. Zhao, D. Wang, Z. Mai, M. Wang, F. Yan, Y. Meng, Y. Xu, G. Xing, Highly stretchable, responsive flexible dual-mode magnetic strain sensor. *Adv. Mater. Technol.* **8**, 2201439 (2023).
25. Q. Zhang, G. Yang, L. Xue, G. Dong, W. Su, M. J. Cui, Z. G. Wang, M. Liu, Z. Zhou, X. Zhang, Ultrasoft and biocompatible magnetic-hydrogel-based strain sensors for wireless passive biomechanical monitoring. *ACS Nano* **16**, 21555–21564 (2022).
26. J. J. Sloots, G. J. Biessels, A. de Luca, J. J. M. Zwanenburg, Strain tensor imaging: Cardiac-induced brain tissue deformation in humans quantified with high-field MRI. *Neuroimage* **236**, 118078 (2021).
27. J.-Y. Bae, G.-S. Hwang, Y.-S. Kim, J. Jeon, M. Chae, J.-W. Kim, S. Lee, S. Kim, S.-H. Lee, S.-G. Choi, J.-Y. Lee, J.-H. Lee, K.-S. Kim, J.-H. Park, W.-J. Lee, Y.-C. Kim, K.-S. Lee, J. Kim, H. Lee, J. K. Hyun, J.-Y. Kim, S.-K. Kang, A biodegradable and self-deployable electronic tent electrode for brain cortex interfacing. *Nat. Electron.* **7**, 815–828 (2024).
28. D.-H. Kim, J. Viventi, J. J. Amsden, J. Xiao, L. Vigeland, Y.-S. Kim, J. A. Blanco, B. Panilaitis, E. S. Frechette, D. Contreras, D. L. Kaplan, F. G. Omenetto, Y. Huang, K.-C. Hwang, M. R. Zakin, B. Litt, J. A. Rogers, Dissolvable films of silk fibroin for ultrathin conformal bio-integrated electronics. *Nat. Mater.* **9**, 511–517 (2010).
29. Y. Roh, M. Kim, S. M. Won, D. Lim, I. Hong, S. Lee, T. Kim, C. Kim, D. Lee, S. Im, G. Lee, D. Kim, D. Shin, D. Gong, B. Kim, S. Kim, S. Kim, H. K. Kim, B.-K. Koo, S. Seo, J.-S. Koh, D. Kang, S. Han, Vital signal sensing and manipulation of a microscale organ with a multifunctional soft gripper. *Sci. Robot.* **6**, eabi6774 (2021).
30. B. Park, J. Kim, D. Kang, C. Jeong, K. S. Kim, J. U. Kim, P. J. Yoo, T.-i. Kim, Dramatically enhanced mechanosensitivity and signal-to-noise ratio of nanoscale crack-based sensors: Effect of crack depth. *Adv. Mater.* **28**, 8130–8137 (2016).
31. T. Kim, I. Hong, Y. Roh, D. Kim, S. Kim, S. Im, C. Kim, K. Jang, S. Kim, M. Kim, J. Park, D. Gong, K. Ahn, J. Lee, G. Lee, H.-S. Lee, J. Kang, J. M. Hong, S. Lee, S. Seo, B.-K. Koo, J.-S. Koh, S. Han, D. Kang, Spider-inspired tunable mechanosensor for biomedical applications. *npj Flex Electron.* **7**, 12 (2023).
32. Y. Huang, Y. Xiang, W. Ren, F. Li, C. Li, T. Yang, Enhancing the sensitivity of crack-based strain sensor assembled by functionalized graphene for human motion detection. *Sci. China Technol. Sci.* **64**, 1805–1813 (2021).
33. D. Kang, P. V. Pikhitsa, Y. W. Choi, C. Lee, S. S. Shin, L. Piao, B. Park, K.-Y. Suh, T.-I. Kim, M. Choi, Ultrasensitive mechanical crack-based sensor inspired by the spider sensory system. *Nature* **516**, 222–226 (2014).
34. T. Sakorikar, M. K. Kavitha, P. Vayalamkuzhi, M. Jaiswal, Thickness-dependent crack propagation in uniaxially strained conducting graphene oxide films on flexible substrates. *Sci. Rep.* **7**, 2598 (2017).
35. J.-H. Lee, J.-Y. Bae, Y.-N. Kim, M. Chae, W.-J. Lee, J. Lee, I.-D. Kim, J. K. Hyun, K.-S. Lee, D. Kang, S.-K. Kang, A fully biodegradable and ultra-sensitive crack-based strain sensor for biomechanical signal monitoring. *Adv. Funct. Mater.* **34**, 2406035 (2024).
36. E. Lee, T. Kim, H. Suh, M. Kim, P. V. Pikhitsa, S. Han, J.-S. Koh, D. Kang, Effect of metal thickness on the sensitivity of crack-based sensors. *Sens.* **18**, 2872 (2018).
37. W. B. Han, G.-J. Ko, K.-G. Lee, D. Kim, J. H. Lee, S. M. Yang, D.-J. Kim, J.-W. Shin, T.-M. Jang, S. Han, H. Zhou, H. Kang, J. H. Lim, K. Rajaram, H. Cheng, Y.-D. Park, S. H. Kim, S.-W. Hwang, Ultra-stretchable and biodegradable elastomers for soft, transient electronics. *Nat. Commun.* **14**, 2263 (2023).
38. W. Hwang, J. Kim, S. Park, T.-H. Kang, S. Kim, K. Lee, M.-G. Lee, R. Kwak, I.-S. Choi, H. Yi, A breathable and stretchable metastructure for a versatile hybrid electronic skin patch with long-term skin comfort. *Adv. Mater. Technol.* **8**, 2200477 (2023).
39. Y. Wang, S. Lee, T. Yokota, H. Wang, Z. Jiang, J. Wang, M. Koizumi, T. Someya, A durable nanomesh on-skin strain gauge for natural skin motion monitoring with minimum mechanical constraints. *Sci. Adv.* **6**, eabb7043 (2020).
40. Y. Jiang, Z. Liu, N. Matsuhisa, D. Qi, W. R. Leow, H. Yang, J. Yu, G. Chen, Y. Liu, C. Wan, Z. Liu, X. Chen, Auxetic mechanical metamaterials to enhance sensitivity of stretchable strain sensors. *Adv. Mater.* **30**, 1706589 (2018).
41. L. E. Hoffman, L. H. S. Van Mierop, Effect of epinephrine on heart rate and arterial blood pressure of the developing chick embryo. *Pediatr. Res.* **5**, 472–477 (1971).
42. J. Wu, D.-S. Zhang, M.-H. Ji, Z. Zhang, J.-J. Yang, Hemodynamic effects of epinephrine in healthy and hemorrhagic shock rats. *Curr. Ther. Res.* **72**, 243–249 (2011).
43. X. Shi, J. Yu, Q. Pan, Y. Lu, L. Li, H. Cao, Impact of total epinephrine dose on long term neurological outcome for cardiac arrest patients: A cohort study. *Front. Pharmacol.* **12**, 580234 (2021).
44. T. Kim, I. Hong, M. Kim, S. Im, Y. Roh, C. Kim, J. Lim, D. Kim, J. Park, S. Lee, D. Lim, J. Cho, S. Huh, S.-U. Jo, C. Kim, J.-S. Koh, S. Han, D. Kang, Ultra-stable and tough bioinspired crack-based tactile sensor for small legged robots. *npj Flex Electron.* **7**, 22 (2023).

45. T. Pepelnjak, A. Karimi, A. Maček, N. Mole, Altering the elastic properties of 3D printed poly-lactic acid (PLA) parts by compressive cyclic loading. *Materials* **13**, 4456 (2020).
46. N. Perumal, S. Sreekantan, Z. A. A. Hamid, A. Rusli, K. Bhubalan, J. N. Appaturi, Effect of plasticizer and compatibilizer on properties of polybutylene adipate-co-terephthalate (PBAT) with acetylated starch. *J. Polym. Environ.* **32**, 289–302 (2024).
47. K. K. Kim, I. Ha, M. Kim, J. Choi, P. Won, S. Jo, S. H. Ko, A deep-learned skin sensor decoding the epicentral human motions. *Nat. Commun.* **11**, 2149 (2020).
48. T. Yang, X. Li, X. Jiang, S. Lin, J. Lao, J. Shi, Z. Zhen, Z. Li, H. Zhu, Structural engineering of gold thin films with channel cracks for ultrasensitive strain sensing. *Mater. Horiz.* **3**, 248–255 (2016).

Acknowledgments
Funding: This work was supported by National Research Foundation of Korea (NRF) grant 2022M3H4A1A04096393 funded by the Ministry of Science and ICT (half supported) and National Research Foundation of Korea (NRF) grant RS-2024-00419269 funded by the Ministry of Science and ICT (half supported). **Author contributions:** Conceptualization: J.-H.L., Y.-N.K., J.L., and S.-K.K. Methodology: J.-H.L., Y.-N.K., and J.L. Investigation: J.-H.L., Y.-N.K., J.L., J.J., J.-Y.B., J.-Y.L., K.-S.K., M.C., H.P., J.-h.K., K.-S.L., J.K., and J.K.H. Visualization: J.-H.L., Y.-N.K., J.L., and S.-K.K. Supervision: S.-K.K. and D.K. Writing the draft: J.-H.L., Y.-N.K., J.L., and S.-K.K. **Competing interests:** The authors declare that they have no competing interests. **Data and materials availability:** All data needed to evaluate the conclusions in the paper are present in the paper and/or the Supplementary Materials.

Submitted 4 September 2024
Accepted 15 November 2024
Published 20 December 2024
10.1126/sciadv.ads9258
