# Supplementary material for: Hypersensitive meta-crack strain sensor for real-time biomedical monitoring
Source: Sci Adv. 2024 Dec 20;10(51):eads9258. doi: 10.1126/sciadv.ads9258 (PMC11661431; doi:10.1126/sciadv.ads9258)
Supplement: Supplementary file 2 — Supplementary Text S1 to S12 Figs. S1 to S38 Tables S1 to S3 References [file sciadv.ads9258_sm.pdf]

Science Advances
AAAS

Supplementary Materials for

Hypersensitive meta-crack strain sensor for real-time biomedical monitoring

Jae-Hwan Lee *et al.*

Corresponding author: Seung-Kyun Kang, kskg7227@snu.ac.kr; Daeshik Kang, dskang@ajou.ac.kr; Jung Keun Hyun, rhhyun@dankook.ac.kr

Sci. Adv. **10**, eads9258 (2024)
DOI: 10.1126/sciadv.ads9258

This PDF file includes:

Supplementary Text S1 to S12
Figs. S1 to S38
Tables S1 to S3
References

Supplementary Text

Supplementary Text 1. Sensor calibration using PZT piezoelectric linear actuator

The resistance variation of the meta-crack sensor (Poisson's ratio -0.9) was analyzed for sensor calibration by the applied voltage-controlled PZT linear actuator inducing controlled tensile strain (tensile displacement rate of 127 nm/V). The sensor (dimension of 29 mm × 3 mm) was attached on top of PZT linear actuator purchased from KEMET corporation (USA) to apply strain quantitatively as shown in Fig. S2. DC voltage ranging from 18 V to 90 V was applied to induce strain on the sensor and the corresponding resistance variation of the sensor was recorded by a digital multi-meter (PXIe-4081, National Instruments, USA). The resistance of the sensor was recorded while applying 18, 36, 54, 72, and 90 V (applied displacement of 2.3, 4.6, 6.9, 9.1, and 11.4 μm, respectively) for 10 seconds each, correspondingly inducing the strains of $7.8\cdot10^{-5}$, $1.6\cdot10^{-4}$, $2.3\cdot10^{-4}$, $3.1\cdot10^{-4}$, and $3.9\cdot10^{-4}$ to the sensor (strain was calculated by dividing the applied displacement by the tensile direction dimension of the sensor of 29 mm). The normalized resistance data (variation of 10^{-3} scale) to applied strain was plotted with standard deviation as shown in Fig. 1C.

The electrical resistance measurement instrument consists of PXIe-4081 DMM (National Instruments, USA) and PXIe-1082 chassis (National Instruments, USA). The DMM has a resolution of 7.5 digits and an accuracy of 12 ppm. Given that the resistance variation of the meta-crack sensor under applied strain (0-1.8%) ranges from tens of kohm to several Mohm, the 7.5-digit resolution is appropriate. The smallest resistance change measured during strain calibration was 0.00072 of normalized resistance, with an accuracy error of 0.000012, yielding an error ratio of just 1.67%.

The temperature of the PZT linear actuator was analyzed with an IR monitoring system (temperature resolution of 0.03 ℃, A655SC, FLIR Systems, USA) while applying 90 V for 5 minutes as shown in Fig. S3. The PZT linear actuator/meta-crack sensor system remained isothermal (temperature variation less than 0.03 ℃) while applying the voltages. The temperature coefficient of resistance (TCR) of the sensor was also analyzed to ensure that the resistance change of the meta-crack sensor was mainly due to induced strain from the PZT linear actuator. The TCR of the sensor was analyzed by recording resistance variation from 25 ℃ to 35 ℃ and the slope of the linear fitted graph was $3.66\cdot10^{-4}$/℃ as shown in Fig. S4. The temperature variation (up to 0.03 ℃) during the operation of the PZT actuator induces normalized resistance variation up to $1.1\cdot10^{-5}$. The normalized resistance variation due to the temperature variation is two orders of magnitude lower than the normalized resistance variation caused by the PZT actuator-induced strain (normalized resistance variation up to $5\cdot10^{-3}$ under $3.9\cdot10^{-4}$ strain). The normalized resistance signal-to-thermal noise ratio of the sensor at $7.8\cdot10^{-5}$ strain exceeds over SNR = 65.6 while maintaining the environment within 0.03 ℃ temperature variation.

To control vibration, a self-standing pneumatic vibration isolation table (Daeil Systems, Korea) was used, maintaining precision leveling with a 0.05 mm amplitude. Airflow was monitored using a flow sensor (NUL-224, NeuLog, Israel) integrated into the setup box, ensuring it remained below 0.001 m/s. Fig. S5 shows the normalized resistance variation under different vibration amplitudes (Fig. S5A) and the baseline monitoring in an air flow rate below 0.001 m/s (Fig. S5B). The normalized resistance noise had a standard deviation of 0.00022 at a 0.1 mm vibration amplitude, and 0.00011 in conditions below 0.05 mm vibration amplitude, 0.03℃ temperature variation, and 0.001 m/s air flow rate.

Supplementary Text 2. Effect of crack morphology on crack opening behavior with diverse Poisson's ratios

The crack gap opening behaviors depending on the Poisson's ratio of substrate were analyzed using finite element analysis (FEA). Substrate with Poisson's ratio of 0.3, typical value of PLA, showed crack gap opening trend varied with the angle (theta) between the tensile-perpendicular direction and the crack edge as in Fig. 1F. The crack with an angle of 10° open faster than the cracks with other angles as the crack orientation is close to tensile-perpendicular direction. As the crack orientation aligns close to the tensile direction, gap widening rate decreases due to lateral contraction by Poisson's deformation. The crack with an angle of 80° showed the gap-narrowing trend which implies the potential of the reconnection of crack edges. Substrate with negative Poisson's ratio (-0.9, the value of the optimized auxetic structure in this paper) showed lower variation of crack gap opening trend comparing with positive Poisson's ratio. Crack gaps increased persistently regardless of the crack orientation due to lateral expansion by Poisson's deformation, which hinders the reconnection of crack edges. Substrate with Poisson's ratio of -0.5 and -0.2 showed intermediate trend between substrate with Poisson's ratio of -0.9 and 0.3, as shown in Fig. S6. The angles of 10, 30, 50° showed the larger gap widening rate with positive Poisson's ratio substrate than the rate with negative Poisson's ratio substrate, which is contrary to the observed trend of electrical resistance change. The difference in the initial straight-line distance between crack edges (D_0) depending on the angle needs to be considered for explaining this discrepancy. Specifically, the straight-line distance between large-angle edges is smaller than that between small-angle edges when considering the initial state (when assuming the length between edges of upper and lower metal pieces in the tensile direction is same in all angles). Therefore, with the smaller initial straight-line distance, the crack gap widening rate of the larger angle is likely the major factor contributing to the observed electrical resistance change.

Supplementary Text 3. Influence of the difference in stiffness between composing layers of the meta-substrate on the distribution of Poisson's ratios

The meta-substrate consists of the base layer, directly deposited with metal film, and the frame layer with specific geometrical auxetic structures. The frame layer with a negative Poisson's ratio should overcome the deformation of the base layer with a positive Poisson's ratio to drive the overall meta-behavior. As the specific geometry of the frame layer is fixed, differences in stiffness between the layers determine the overall deformation. Accordingly, variations in strain transfer to the metal film arise depending on the thickness and elastic modulus ratios of both layers.

Figs. S9 and S10 show the distribution of ε_{yy} and Poisson′s ratio on the surface of the base layer under various ratios of the elastic modulus and thickness between the frame and base layers. The analysis of elastic modulus ratios involved setting these ratios at 1, 10, 10^2, 10^3, and 10^4, while keeping the thickness ratio fixed at 1 (Fig. S9). Similarly, for thickness ratios, we used values of 10^{-1}, 3^{-1}, 1, 3, and 10, with the elastic modulus ratio held constant at 1 (Fig. S10). A fixed applied tensile strain (ε_{xx}) was 0.02. Regarding the elastic modulus, a negative Poisson's ratio distribution emerged when the frame-to-base ratio reached 10, with saturation tendencies beginning at 10^2 and stabilizing at 10^3. For thickness, the negative Poisson's ratio distribution began to appear at a ratio of 10, with expansion observed from a ratio of 30.

Figs. S11 and S12 show the distribution of ε_{yy} and Poisson's ratio on the surface of the base layer for elastic modulus ratios of 10^2 and 10^4 between the frame and base layers, with thickness

ratios of 1/3, 1, and 3. The applied tensile strain ε_{xx} was 0.02. In both elastic modulus ratio cases, the negative Poisson's ratio distribution began to saturate when the thickness ratio was 1/3, and the distribution remained stable from a ratio of 1 to 3.

In our case, the mainly reported meta-substrate has an elastic modulus ratio of 148.94 and a thickness ratio of 0.33 between the frame and base (PLA frame: 3.5 GPa, 10 μm; PBAT base: 23.5 MPa, 30 μm) (*45*, *46*). This suggests that a negative Poisson's ratio distribution, at least equivalent to the level shown in Fig. S11A, has been transferred to the metal film.

Supplementary Text 4. Buckling effect in the meta-substrate by the asymmetrical composition

The buckling of the meta-substrate could occur attributed to its asymmetrical composition, including the base and frame layers. Fig. S13 analyzes the deformation of the representative meta-substrate in both free-standing and target-attached states using FEA to assess the buckling effect. The employed auxetic structure was a horizontal re-entrant pattern, and the applied tensile strain ε_{xx} was 0.02. The precondition of z-axis constraint was set on the surface of the frame for a target-attached state. In the free-standing state, maximum z-axis deflection reached tens of micrometers, while in the target-attached state, it was limited to 2 micrometers. The y-axis strain distribution showed poor periodicity intensifying toward the lateral edges in the free-standing state but became periodically uniform in the target-attached state depending on the auxetic patterns. The non-uniform distribution of z-axis displacements and y-axis strains in the free-standing state indicates the occurrence of buckling effect. Meanwhile, the periodically uniform y-axis strain distribution in the target-attached state suggests negligible occurrence of buckling effect, showing only partial z-axis shrinkage resulted from the implementation of negative Poisson's ratio in applications.

Supplementary Text 5. Theoretical modeling for the resistance variation of crack sensors with various Poisson's ratios

The previous theoretical modeling proposed based on probabilistic strain-conductance behavior of conductive cracked film with random step-like crack edges shows aspects of decreasing conductance using a log-normal probability distribution function in different rate by applied strain depending on disconnection rate and reconnection occurrence of the crack edges. (*31, 33*) The resistance of the crack based sensor can be calculated using an equation (*31*):

$$R = \left(\frac{1}{2}\left(1 - erf\left(\frac{ln\left(\frac{k\varepsilon}{\delta_0}\right)}{\mu}\right)\right)\right)^{-1} \qquad (1)$$

The parameter δ_0 (initial crack gap size) is measured by SEM imaging and μ (deviation of probability distribution) is the fitting parameter based on the theoretical modeling (measured and fitted parameters of meta-crack sensors with diverse Poisson's ratios in Supplementary table 2). The proportionality factor k indicates the rate of crack gap increase by applied strain (*31*) consequently related to the Poisson's ratio deciding the disconnection rate of the crack edges. The lower value of negative Poisson's ratio induces the faster disconnection of the crack edges under applied strain, resulting in the higher k value of the sensor. The fitting results show inverse relation between Poisson's ratio and the factor k, indicating that the lower Poisson's ratio induces the faster

crack gap widening under same applied strain (Supplementary table 2). The inverse relation between Poisson's ratio and proportionality factor k showed a linear fitting result with a line formula:

$$k = a + b\nu \quad (2)$$

The fitting parameters a and b is 38.93 and -108.77.

Supplementary Text 6. FEA for calculation of Poisson's ratio for various parallel circuited sensors

Deformation of parallel circuited sensor was analyzed based on 3D FEA simulation by modeling with attachment of 10 μm PLA on 30 μm PBAT with elastic modulus of 1 MPa and Poisson's ratio of 0.4. Interface of two layers was constrained by applying tie option. A side of PLA-PBAT film was stretched in positive x direction when the other side was fixed as experiment performed. Fig. S17 represent the uniformly patterned strain distribution in structures of parallel circuit combining auxetic and plane PLA region, which was basis of Poisson's ratio shown in Fig. 3. Positive ε_{yy}, shades of red color, distributes through auxetic structures shown as Fig. S17 (A and B), while lateral contraction, negative ε_{yy} colored in shades of blue color, resulting from Poisson's effect is showed in plane structured area. Strain variation in ε_{xx} was shown due to the difference in stiffness, when overall area showed positive ε_{xx} as applied as shown in Fig. S17 (C and D). Grip regions were applied ε_{xx} of 0~2%, whereas plane region of parallel structured were applied ε_{xx} of 2~3%. Wide variation in ε_{xx} was measured inside of unit structure due to the opening of re-entrant auxetic structure.

Supplementary Text 7. Modified theoretical modeling for interpreting the resistance variation of various parallel circuited sensors

The resistance variation of the parallel circuited sensor can be calculated by simplifying each parallel element as an independent resistor (resistance of the parallel element is calculated with an equation (1) of the previous theoretical modeling) and assuming a parallel resistor circuit. The resistance of the parallel resistor circuit can be calculated using an equation:

$$R = \frac{1}{\frac{1}{R_1} + \frac{1}{R_2} + \frac{1}{R_3} + \cdots + \frac{1}{R_n}} \quad (3)$$

where R_1, R_2, R_3, ⋯, R_n are the resistance of each parallel resistor. Meanwhile, the actual parallel circuited sensor experiences deformation interference due to the structural attachment of each parallel element to a single substrate unlike the ideal assumption that each element operates independently. Therefore, an adjustment is needed for the modified theoretical modeling based on the parallel resistor circuit by adding a fitting parameter to account for the interference effect. The resistance of the parallel circuited sensor can be estimated using an equation:

$$R = \frac{1}{\frac{i_{\nu 1}}{R_{\nu 1}} + \frac{i_{\nu 2}}{R_{\nu 2}} + \frac{i_{\nu 3}}{R_{\nu 3}} + \cdots + \frac{i_{\nu n}}{R_{\nu n}}} \quad (4)$$

where $R_{\nu n}$ is obtained using equation (1) with certain Poisson's ratio ν and n is the number of resistor elements of the parallel circuit. The value $i_{\nu n}$ is the fitting parameter to account for the interference effect, meaning an interfering factor for effect of certain Poisson's ratio ν on overall resistance calculation. In addition, the parallel circuited sensor with combination of several

Poisson's ratios shows higher value of $i_{\nu n}$ with lower composition ratio of the corresponding Poisson's ratio.

Fig. 3E shows the comparison of normalized resistances of the representative parallel circuited sensors with different substrates. The theoretical modeling is possible using simplified formula based on (4):

$$R = \frac{1}{\frac{i_{\nu-0.9}}{R_{\nu-0.9}} + \frac{i_{\nu 0.3}}{R_{\nu 0.3}}} \quad (5)$$

when $R_{\nu-0.9}$ and $R_{\nu 0.3}$ indicate the resistances of sensors on the substrates having each Poisson's ratio of -0.9 and 0.3, respectively. $i_{\nu-0.9}$ and $i_{\nu 0.3}$ are the fitting parameters for the Poisson's ratios of -0.9 and 0.3 (Supplementary table 3). The fitting results using the equation (5) for the representative parallel circuited sensors with various composition ratios in Fig. 3E indicate that higher fraction of the negative Poisson's ratio induces higher resistance change rate with lower interfering factor $i_{\nu-0.9}$, consequently giving higher GF change rate under the critical strain of the negative Poisson's ratio (Supplementary Table 3). Higher absolute value of the negative Poisson's ratio also induces higher rate of resistance change, raising overall GF values in all sensing range. Calculated values of fitted normalized resistance match well up to the experimental gauge factor values. Some discrepancy occurred in the sensor with a 2:1 ratio (blue), showing the GF decrease in the large strain region (Fig. 3D). The reason of the deviation from theoretical fitting is that the parallel arrangement of the auxetic structure did not guarantee fully independent strain distribution at each location. The normalized resistance behavior in Fig. 3E should be examined to understand the GF variation under applied strain. All parallel-circuited meta-crack sensors showed increasing resistance with applied strain, but the 2:1 ratio sensor exhibited a reduced slope of the resistance increase beyond 0.8% applied strain. The cause can be inferred from Fig. S17B, which shows the ε_{yy} distribution on the substrate surface of the 2:1 ratio sensor. The area experiencing y-axis compression around the auxetic structure increased as the fraction of negative Poisson's ratio in the parallel-circuited meta-substrate increased. This led to a wider unexpected positive Poisson's ratio distribution on the substrate surface, affecting the large strain range where meta-crack opening has less impact, resulting in a smaller slope than theoretically expected for resistance increase in that region. As a result, the GF value showed a slight decrease in the large strain region, while the normalized resistance consistently increased with applied strain.

Supplementary Text 8. FEA based estimation of fungi touching-induced force and deflection on pressure sensing device

The force induced by fungi demonstrated in Fig. 4 (D and E) was roughly analyzed based on FEA. The fungi-induced force was simplified as a point load applied on the PBAT base matrix, with the location of exact center of the trench. The touching force and vertical displacement of the deflection were analyzed when the strain applied on the location of the meta-crack sensor was reached as $6.3 \cdot 10^{-5}$. The analyzed strain level exceeded the noise level of strain in the experiment (Fig. 4E and Fig. S23), showing that the minimum force and displacement level measurable with the pressure sensor can be estimated smaller than 3 μN and 2 μm based on the FEA results.

Supplementary Text 9. Effect of mesh-structured substrate on as-is target deformation

The substrate of sensor constrains the deformation of the target, disrupting the as-is deformation when the high elastic modulus substrate is attached on the target with a low elastic modulus. The greater the difference in elastic modulus, the stronger this constraint becomes. However, the effective modulus significantly decreases compared to the original modulus by the mesh-structured substrate to reduce its volumetric stiffness (*38*), allowing it to more closely match the as-is deformation of the target. In this case, the accurate signal monitoring is possible without the attenuation of the target deformation. Relatively high Young's modulus (over 100 MPa) of the previously used plane substrate hinders as-is deformation of soft materials, such as dura mater (under 50 MPa), induced by the outward pressure change of vessels. The high compliance deformation of the soft materials is possible with a low volumetric stiffness by attaching the mesh-structured substrate, exhibiting readily deformation in the z-axis direction. The FEA results in Fig. S26 show the 2.38-fold amplified strain induced immediately above the vessel with the mesh-structured substrate compared to the plane substrate based on a brain tissue model. The degree of amplification of the induced strain changes based on the design factors of the mesh structure. Control of the factors indicating width and length dimension of bridge in the mesh configuration provide the results that the shorter width and longer length lead to the higher amplification of the induced strain for the same exerted pressure change.

Optimization of mesh structure for maximizing strain sensitivity was performed based on FEA simulation prior to in-vivo test. Mesh-structured substrate attached on durameter with thickness of 0.27 mm, covering brain tissues with a blood vessel was modeled. Linear elastic model was used with elastic modulus of 40 MPa and 1 kPa, and Poisson's ratio of 0.45 and 0.49 for durameter and brain tissue, respectively. Geometry of the mesh-structured substrate was modified by varying the width (0.5, 1.0 and 1.5 mm) and length (2, 4 and 6 mm) of mesh structure. The FEA results in Fig. S25B show that the strains transferred to the top surface of the substrates from skin get close to the level of strain developed on bare-skin when the mesh length increases and the width decreases. Geometry of the mesh-structured substrate was determined to be width of 0.5 mm and length of 4 mm, considering feasible size of the individual meta-crack sensor based on the fabrication process and the intervals between the target vessels. The strain analyzed on the mesh-structured substrate with the optimized geometry, with the significantly decreased elastic modulus from 855 to 0.19 MPa (Fig. S25C), showed 79.3% level compared to the strain applied on bare dura mater without sensor substrate (Fig. S26). The results indicate a significant advantage comparing with the plane substrate in terms of strain sensitivity.

Supplementary Text 10. Sensing and recording interfaces of meta-mesh array sensors in canine brain model

The cerebrovascular pulse sensing/recording was conducted with various types of meta-mesh array sensors as shown in Figs. S27 and S32. All types of sensors were placed on the same beagle brain with same position depicted as a blue circle. Ag nanowire sensor and plane crack sensor were used as a sensitivity comparison to our meta-mesh crack sensor. The resistance data of honeycomb-like positioned array sensors were recorded with multi-channel resistance recording device integrated with a multi-channel switch (PXIe-2526, National Instruments, USA), a digital multi-meter (PXIe-4081, National Instruments, USA), and a PXIe-1082 chassis (National Instruments, USA). Meta-crack sensor with mesh-substrate showed the highest sensitivity and could visualize the cerebral vessels position while the Ag nanowire sensor with the same patterned

substrate was hard to differentiate each positions with the resistance data (Fig. S30). Meta-crack sensor without the mesh pattern showed the second highest sensitivity which implies that the meta-structure effect is more influential than the mesh-substrate effect (Fig. S30).

Supplementary Text 11. Cerebrovascular surface strain monitoring in rat and canine brain model

The meta-crack sensor array (Poisson's ratio of -0.9, 2x2 array) with mesh structured substrate was placed on a rat brain for localized cerebral vein pulse sensing as shown in Fig. S28. The 2x2 array meta-crack sensors were placed at positions depicted as a number on Fig. S28A. The sensor at location 1 was placed on top of the cerebral vessel while sensors at location 2,3,4 were placed where no vessel was visible. The sensor placed on location 1 showed ~0.5% increase in normalized resistance implying that the vessel pulse induce ~10^{-4} strain which coincides with the strain analyzed by previous study (*26*). In-vivo strain sensing was also conducted on a beagle brain as shown in Fig. S29. The sensor was placed in the blue circle depicted in Fig. S29A. The blood pulse rate was ~50 BPM while resistance recording with a digital multi-meter as shown in Fig. S29 (B and C). The resistance data showed periodical cycle with 1.2 s period which correlates with the actual blood pulse rate (*35*). The overall fluctuation of the resistance with larger period may be caused by inhalation/exhalation.

Supplementary Text 12. Demonstration of NFC based wireless meta-crack sensor

The integration of simple wireless communication system-on-chip (SoC) with flexible patch type meta-crack sensor enables on-board hyper-sensitive surface strain sensing and wireless data transmission on arbitrary target. The wireless, battery-free operation of the integrated SoC-sensor module is available exploiting NFC chipsets and interfacing circuits (circuit design CAD image in Fig. S33). Fig. S35 shows illustration and block diagram of the wireless sensing device including the sensor, NFC components, and coil antenna for wireless power transfer and corresponding data communication. Near-field magnetic inductive coupling between NFC reader and receiver coil delivers power at a frequency of 13.56 MHz to NFC interface. The integrated microcontroller and transponder transfer the resistance based strain sensing data toward NFC reader in series. The overall flexible wireless sensing device attached on any curved targets provides real-time strain monitoring data from a distance. Fig. S34 shows photograph of the device presenting overall dimension within 20 mm, compared to a penny.

Fig. 5 (F and G) and Figs. S35 and S37 summarize the results of simple NFC SoC based on-board wireless, battery-free vascular pulse monitoring on dura mater above the vessel with blood pressure elevating process. The wireless sensing device integrated with the meta-crack sensor and NFC interface is attached on the vessel after opening the skull. Conventional suture threads hold the operated scalp closed, and then the NFC reader is located above the head for supplying power through the magnetic inductive coupling (inset of Fig. 5E). Fig. 5F shows real-time monitoring of normal blood flowing state and following excited state after epinephrine injection at a vein beneath the skin of the leg (photograph in Fig. S37) and recovery to the normal state after the end of the drug effect, expressed as normalized resistance changes (Fig. 5G and Fig. S36). The normalized resistance values are converted from recorded data set of ADC values in the form of hexadecimal number recorded in the NFC chip. The proper amount of the epinephrine (4 μg/kg, 10 kg for the operated beagle) induces an increased vascular smooth muscle contraction

tightening the blood vessel, consequently elevating the vascular pressure within certain time. (*35*, *41*-*43*) The excited state lasted over 2 minutes with the gradual pressure increase (*35*, *42*, *43*), and then returned to the normal state showing stable pulse rate induced by normal blood flow with the approximate value of 50 BPM in Fig. S36B (*35*).

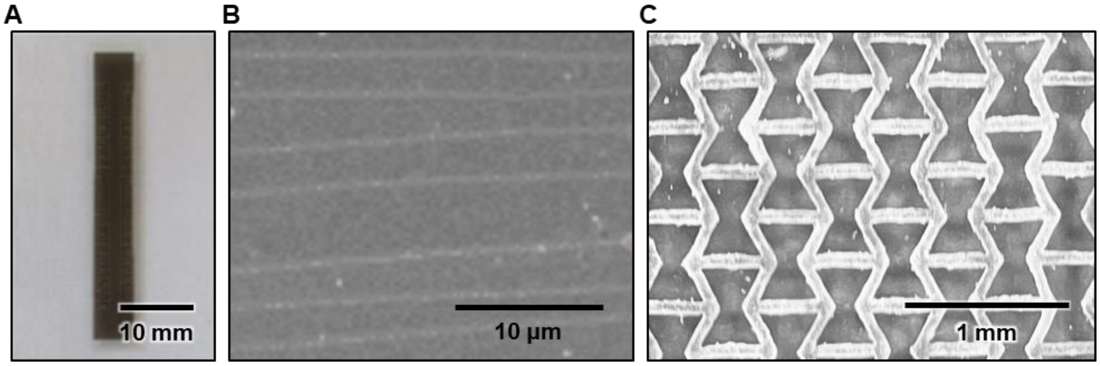

Fig. S1. Images of the meta-crack sensor and its constituent elements. (**A**) Photograph of the overall meta-crack sensor. (**B**) SEM image of the enlarged meta-crack sensor with several micrometers-interval nanoscale cracks. (**C**) Optical microscope image of auxetic structures of the meta-substrate.

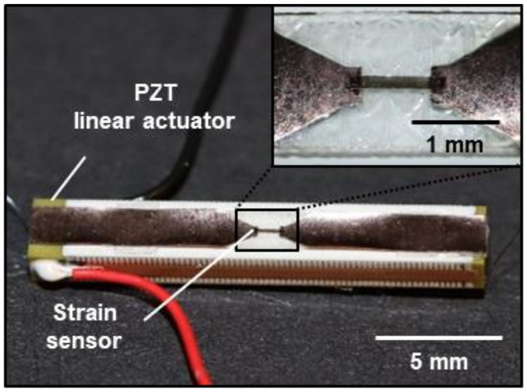

Fig. S2. PZT linear actuator-based calibration of the meta-crack sensor for extremely low strain sensing.

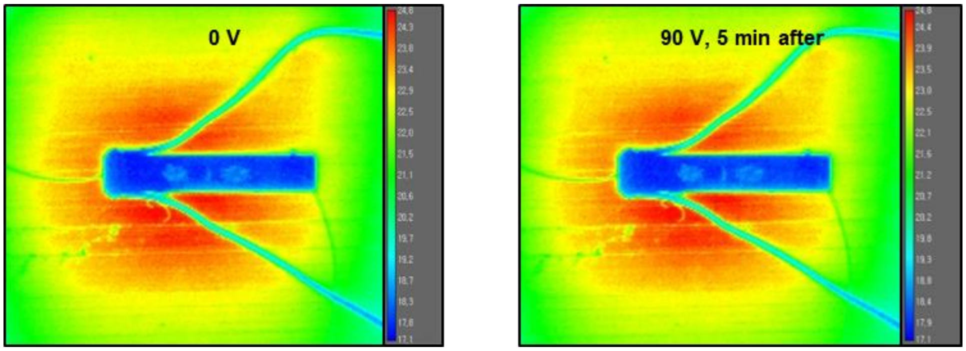

Fig. S3. Thermal distribution mapping during PZT linear actuator-induced calibration of the meta-crack sensor.

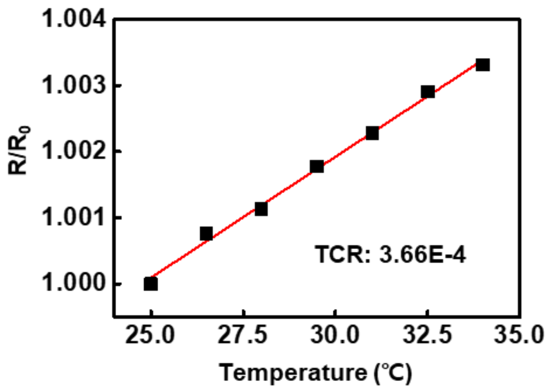

Fig. S4. Temperature calibration curve of the meta-crack sensor.

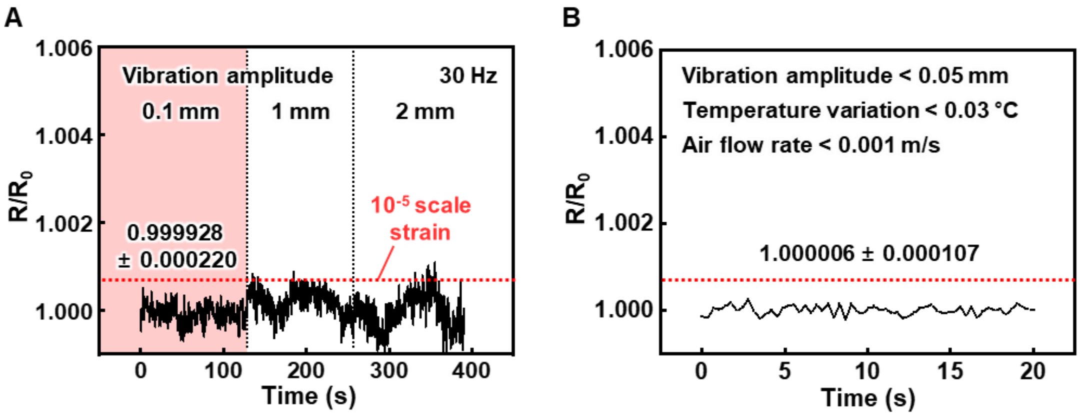

Fig. S5. Variation of normalized resistance of the meta-crack sensor induced by the influence of the environmental factors. (**A**) Influence of vibration amplitude varying from 0.1 to 2 mm under the frequency of 30 Hz. Red dot line shows the sensing normalized resistance induced by the $7.8 \cdot 10^{-5}$ strain. (**B**) Variation of base normalized resistance under control of vibration amplitude, temperature, and air flow rate of under 0.05 mm, 0.03℃, and 0.001 m/s, respectively.

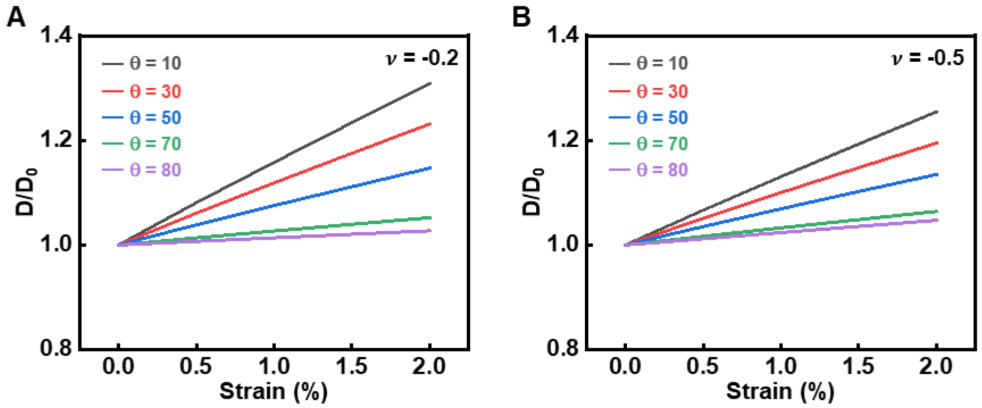

Fig. S6. Crack gap widening behavior under 0-2·10^{-2} strain range with Poisson's ratios of (**A**) -0.2 and (**B**) -0.5.

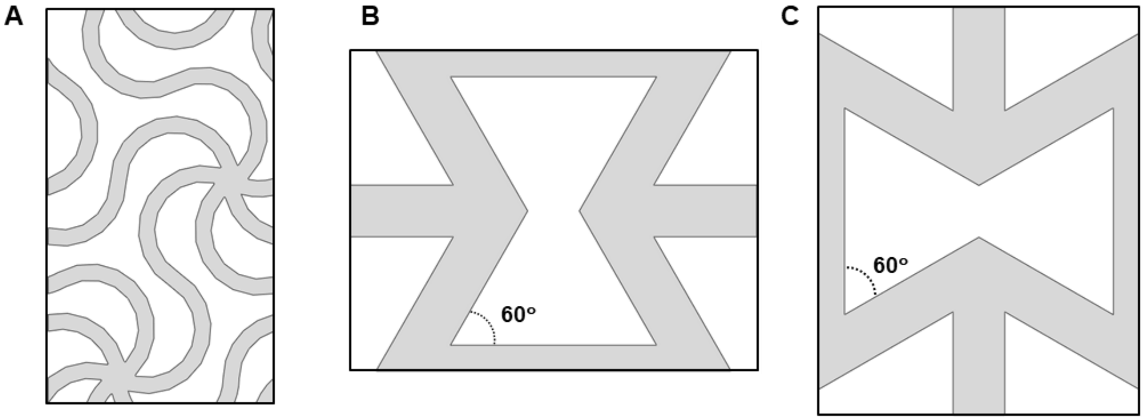

Fig. S7. Unit cell geometries of (**A**) rotachiral, (**B**) vertical re-entrant, and (**C**) horizontal re-entrant auxetic structures.

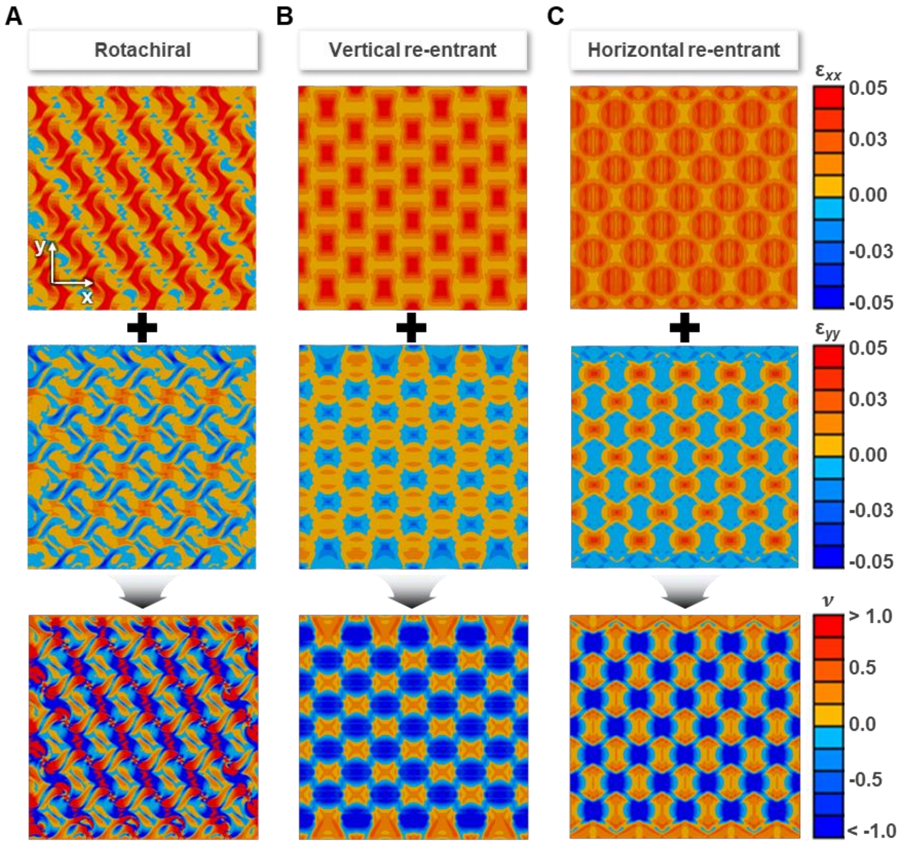

Fig. S8. Non-uniform distribution of x-, y-axis strains and the calculated Poisson's ratios on the surface of the meta-substrates under applied ε_{xx} of 0.02 with each auxetic structure of (**A**) rotachiral, (**B**) vertical re-entrant, and (**C**) horizontal re-entrant pattern.

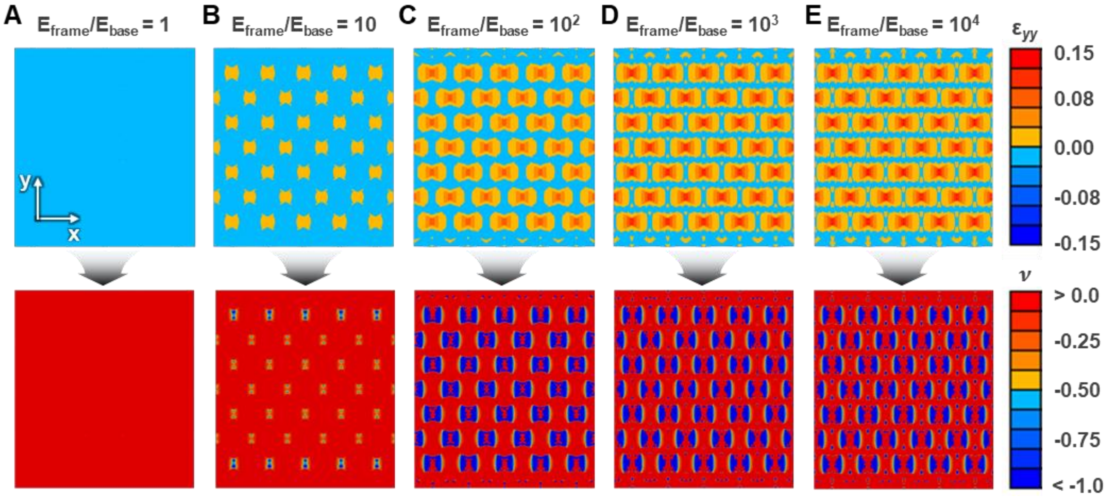

Fig. S9. Y-axis strain and Poisson's ratio distribution on the surface of the meta-substrate under applied ε_{xx} of 0.02 with the same thickness between base and frame layers. Elastic modulus ratios of (**A**) 1, (**B**) 10, (**C**) 10^2, (**D**) 10^3, and (**E**) 10^4 for each case.

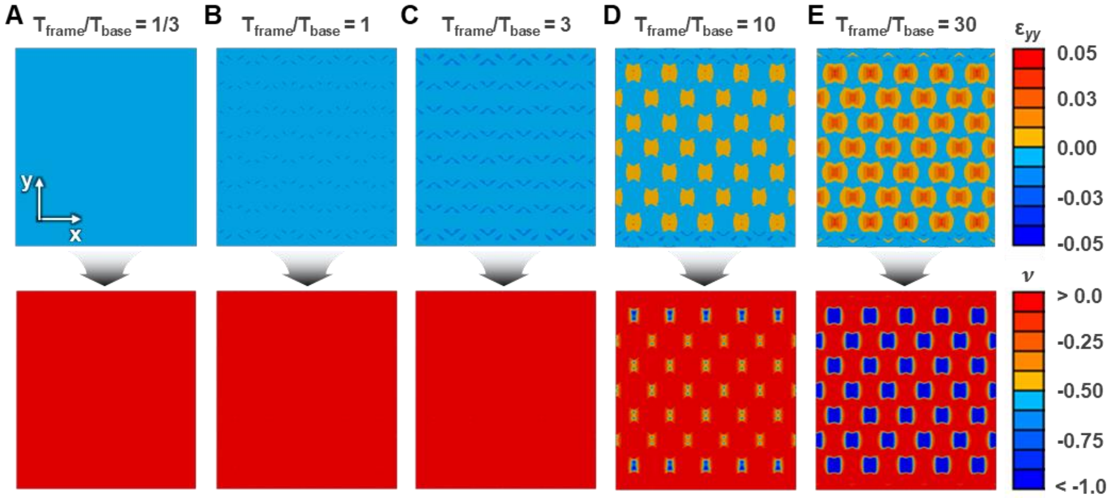

Fig. S10. Y-axis strain and Poisson's ratio distribution on the surface of the meta-substrate under applied ε_{xx} of 0.02 with the same elastic modulus between base and frame layers. Thickness ratios of (**A**) 1/10, (**B**) 1/3, (**C**) 1, (**D**) 3, and (**E**) 10 for each case.

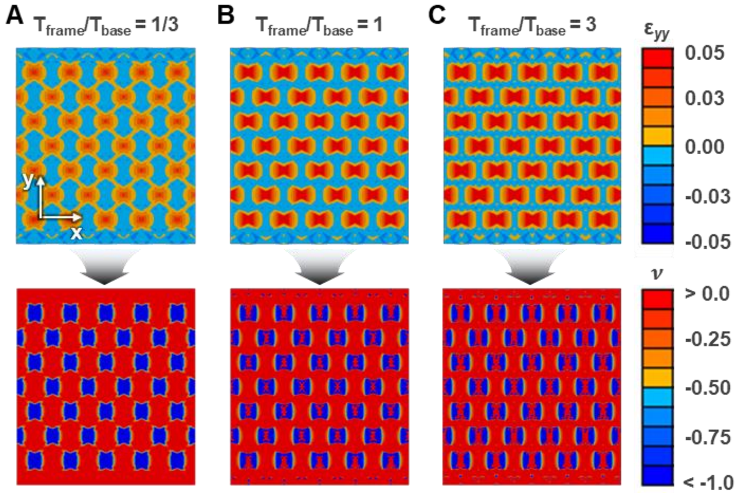

Fig. S11. Y-axis strain and Poisson's ratio distribution on the surface of the meta-substrate under applied ε_{xx} of 0.02 with the elastic modulus ratio of 10^2 (frame/base). Thickness ratios of (**A**) 1/3, (**B**) 1, and (**C**) 3 for each case.

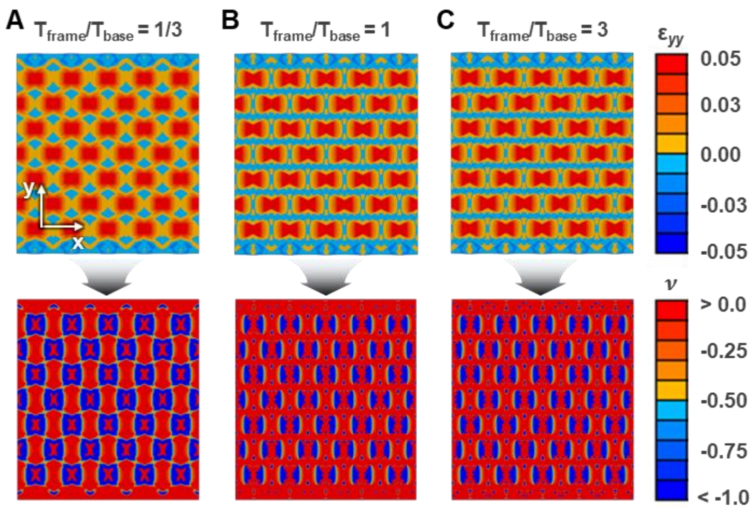

Fig. S12. Y-axis strain and Poisson's ratio distribution on the surface of the meta-substrate under applied ε_{xx} of 0.02 with the elastic modulus ratio of 10^4 (frame/base). Thickness ratios of (**A**) 1/3, (**B**) 1, and (**C**) 3 for each case.

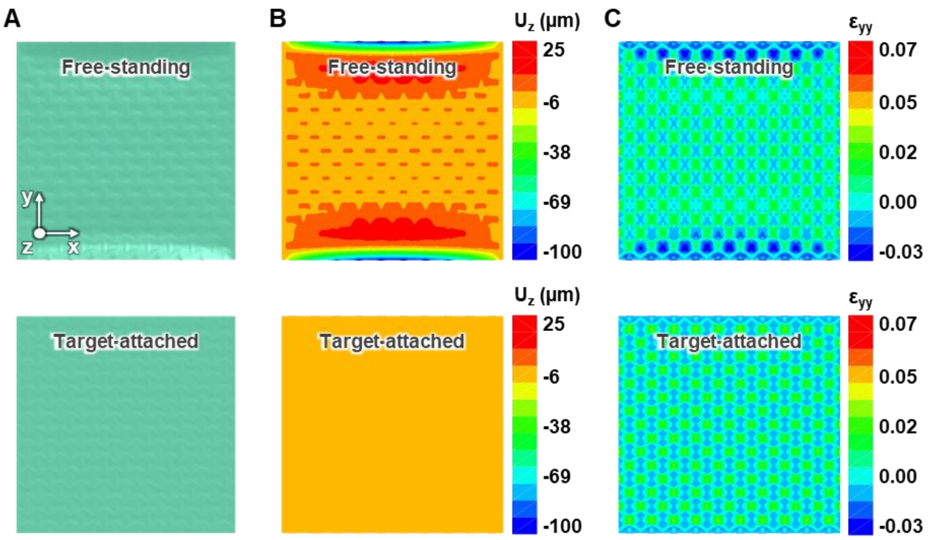

Fig. S13. Degree of buckling with free-standing and target attached state of the meta-substrate under applied ε_{xx} of 0.02. (**A**) Top view geometry of the meta-substrate under each state. (**B**) Comparison of the z-axis deflection distribution between the states. (**C**) Comparison of the y-axis strain distribution between the states.

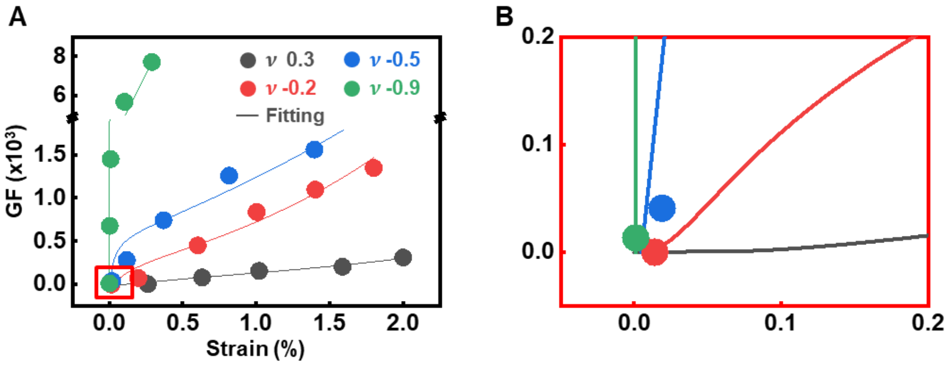

Fig. S14. Variation of GF for the sensors with various Poisson's ratios (0.3, -0.2, -0.5, and -0.9), fitted with the theoretical model. The GFs through (**A**) each overall strain sensing range and (**B**) enlarged strain and GF ranges.

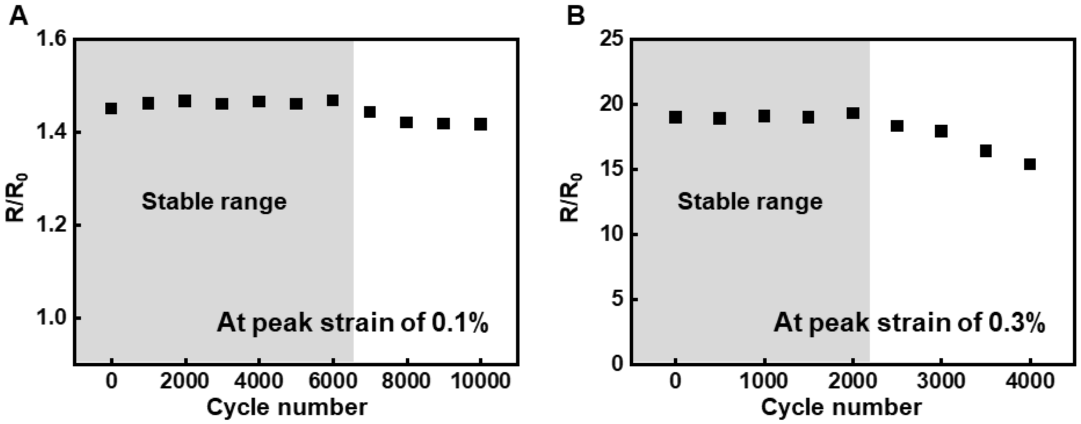

Fig. S15. Long cyclic durability test of the meta-crack sensor. (**A**) 10,000 loading-unloading cycles with a peak strain of 0.1%. (**B**) 4,000 loading-unloading cycles with a peak strain of 0.3%.

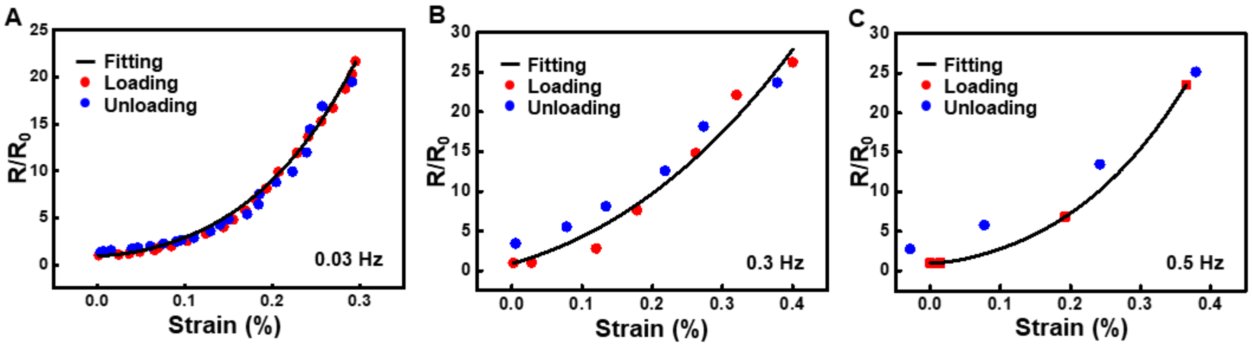

Fig. S16. Hysteresis behavior of the meta-crack sensor with diverse loading-unloading frequencies. Tensile frequencies of (**A**) 0.03 Hz, (**B**) 0.3 Hz, and (**C**) 0.5 Hz.

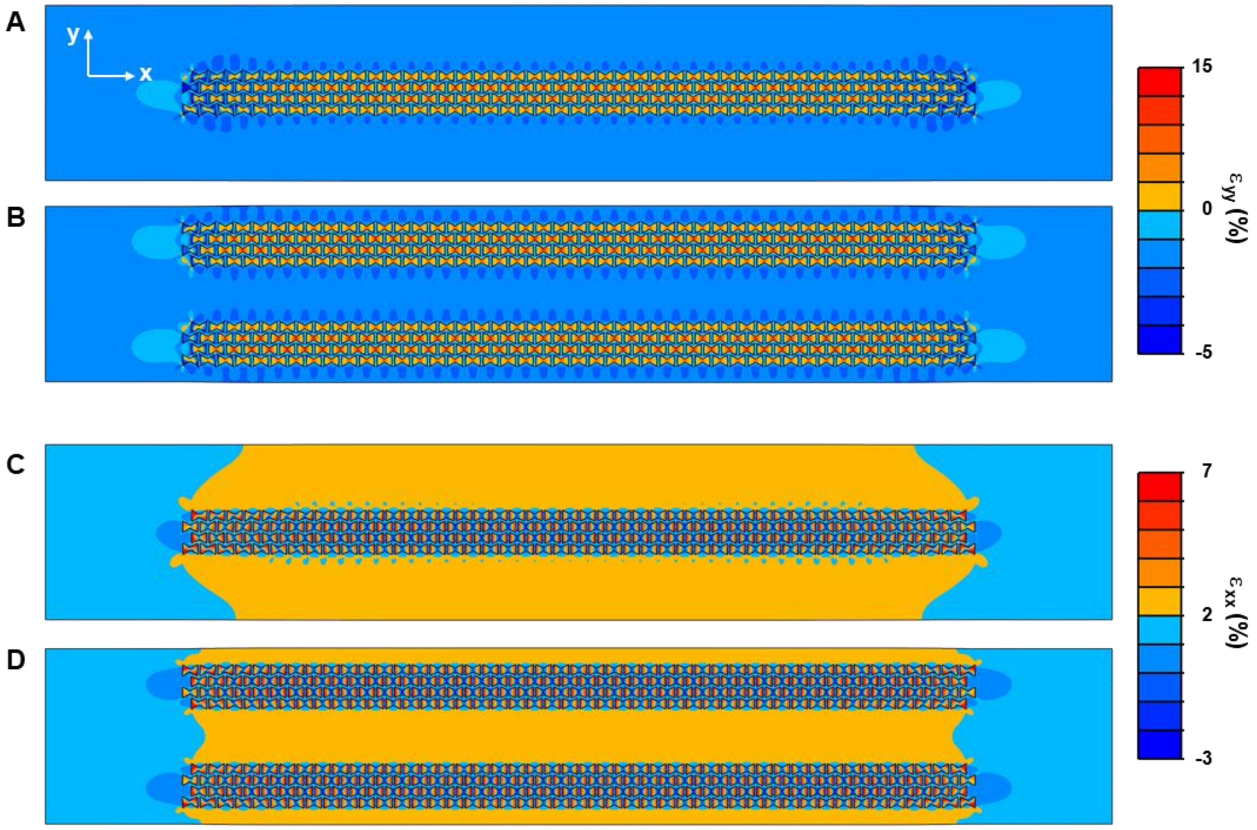

Fig. S17. Strain distribution of parallel circuited structure with combination of plane and horizontal re-entrant auxetic structures. (**A**) Axial-strain distribution of 1:2 (plane:auxetic) substrate. (**B**) Axial-strain of 2:1 (plane:auxetic) substrate. (**C**) Lateral-strain of 1:2 (plane:auxetic) substrate. (**D**) Lateral-strain of 2:1 (plane:auxetic) substrate.

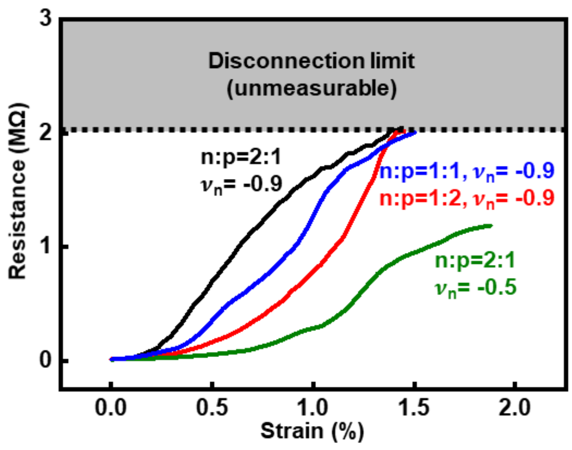

Fig. S18. Resistance variation of the representative parallel circuited sensors under the disconnection limit.

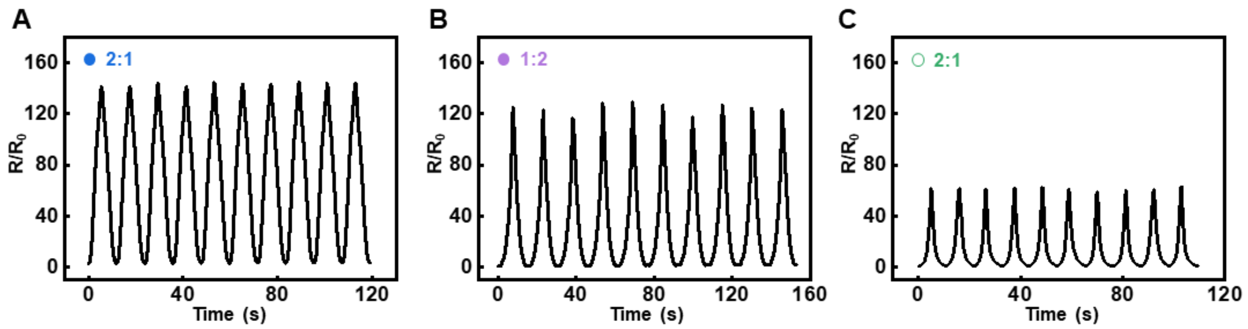

Fig. S19. Confirmation of sensing repeatability of the representative parallel circuited sensors up to 1.5% strain. Composition ratio of (**A**) 2:1 ($\boldsymbol{\nu}$-0.9:$\boldsymbol{\nu}$0.3), (**B**) 1:2 ($\boldsymbol{\nu}$-0.9:$\boldsymbol{\nu}$0.3), and (**C**) 2:1 ($\boldsymbol{\nu}$-0.5:$\boldsymbol{\nu}$0.3).

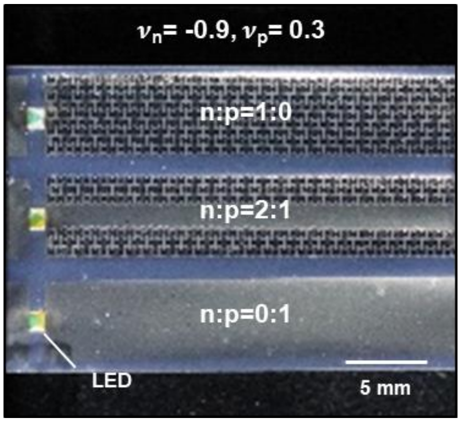

Fig. S20. Photograph of backside sensor array for tensile demonstration with LED including high critical strain with high GF sensor.

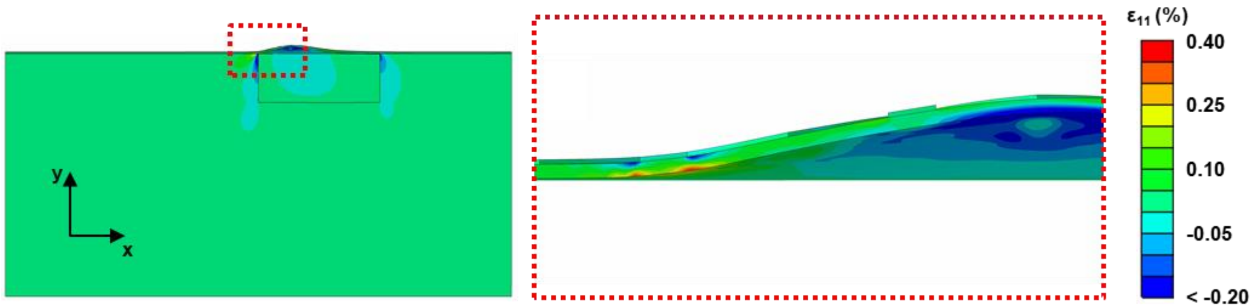

Fig. S21. FEA results simulating deformation of the meta-crack sensor on the PDMS trench by a beneath heat source.

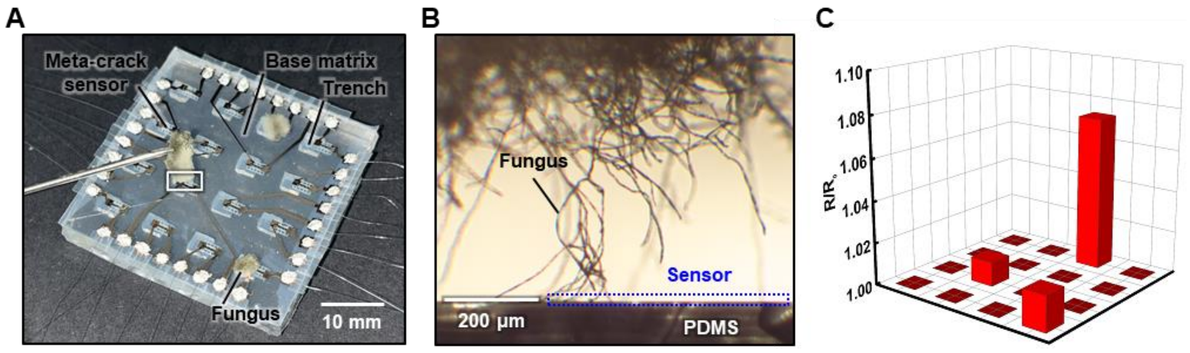

Fig. S22. Demonstration of the pressure sensor array with fungi pieces. (**A**) Photograph of pressure sensor array integrated with the meta-crack sensor array on PDMS trench with fungi pieces. (**B**) Enlarged photograph of the fungi piece touching the substrate of the meta-crack sensor. (**C**) Distribution of the pressure sensing with the meta-crack sensor array-integrated pressure sensor array with the fungi pieces.

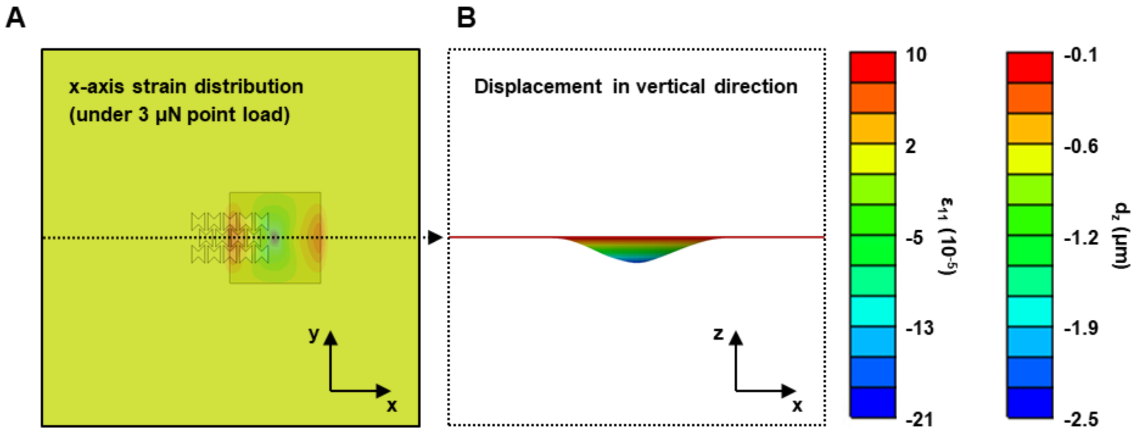

Fig. S23. FEA results simulating deformation of the pressure sensor integrated with the meta-crack sensor on PDMS trench by fungi growing-induced touching. (**A**) Strain distribution in x-axis direction with a point load in the middle location of the trench on the substrate. (**B**) Displacement distribution in z-axis direction.

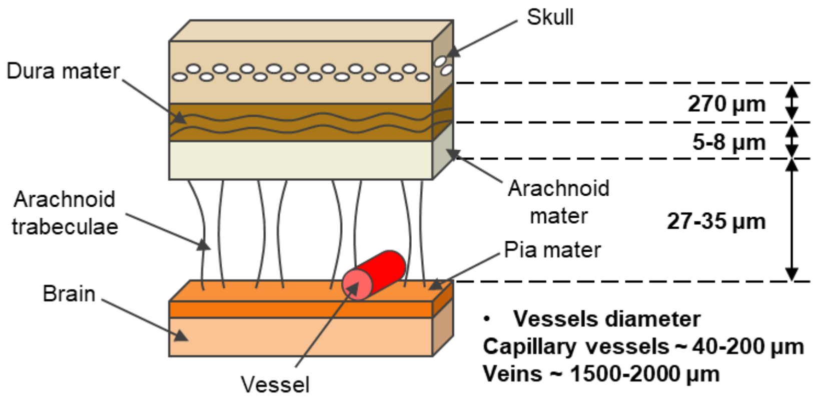

Fig. S24. Anatomic composition of the sensing target (brain model) below the sensing position.

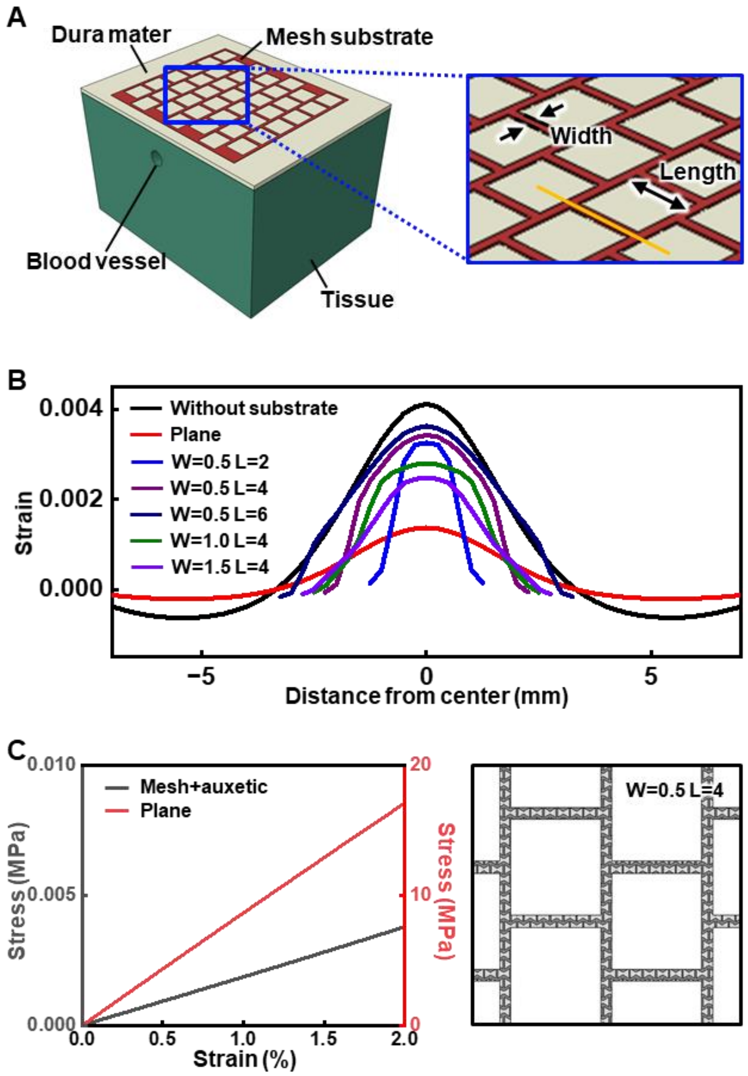

Fig. S25. FEA for comparison of target deformation compliance with diverse dimension mesh-structured substrates. (**A**) Illustration of mesh-structured substrate attached on the dura mater with a beneath blood vessel. Enlarged view shows the geometrical parameters including width and length. (**B**) Strain distribution on the surface line of the mesh-structured substrate right above the blood vessel in vessel-perpendicular direction (yellow line in a), with diverse widths and lengths, compared with states of without substrate and plane substrate. (**C**) FEA results for the comparison of elastic modulus of plane substrate and effective elastic modulus of the optimized mesh- and auxetic-structured substrate. Illustration shows the optimized mesh- and auxetic-structured substrate (width, 0.5 mm; length, 4 mm).

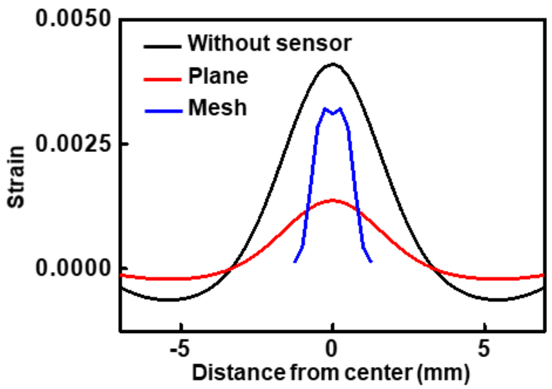

Fig. S26. Effect of the optimized mesh-structured substrate on as-is deformation of the sensing target (brain model) based on FEA.

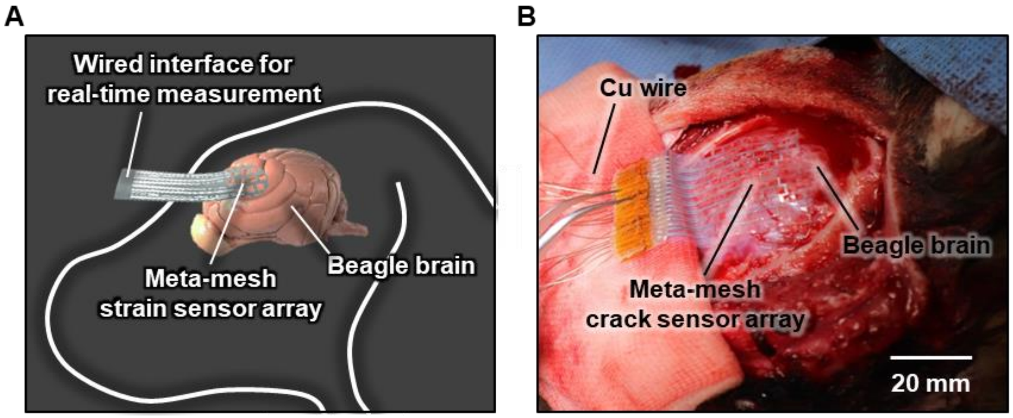

Fig. S27. Experimental setup images on a canine brain model by (**A**) illustration and (**B**) photograph.

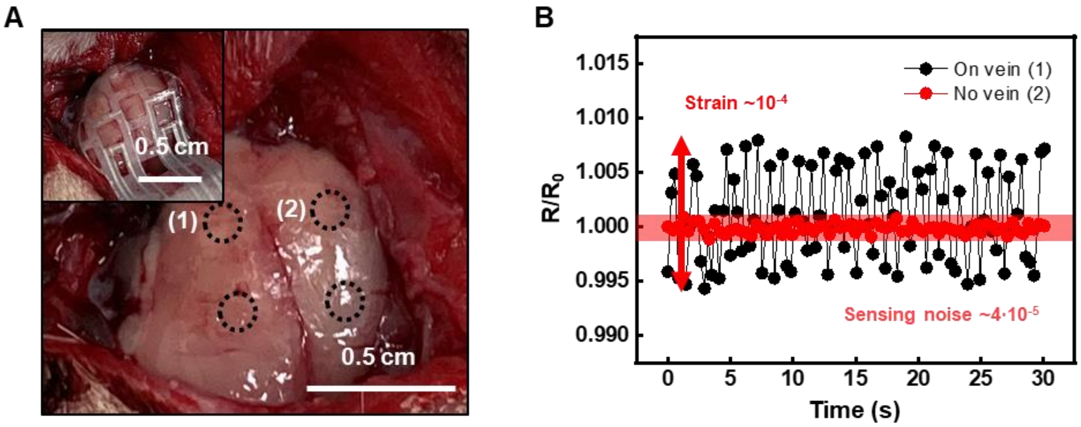

Fig. S28. ***In-vivo*** **demonstration of cerebrovascular dynamics monitoring on a rat brain model.** (**A**) Photographs of the rat brain model with the meta-crack sensor array (2 by 2, inset). (**B**) Monitoring results of cerebrovascular (venous) pressure using the meta-crack sensor array on the rat brain model.

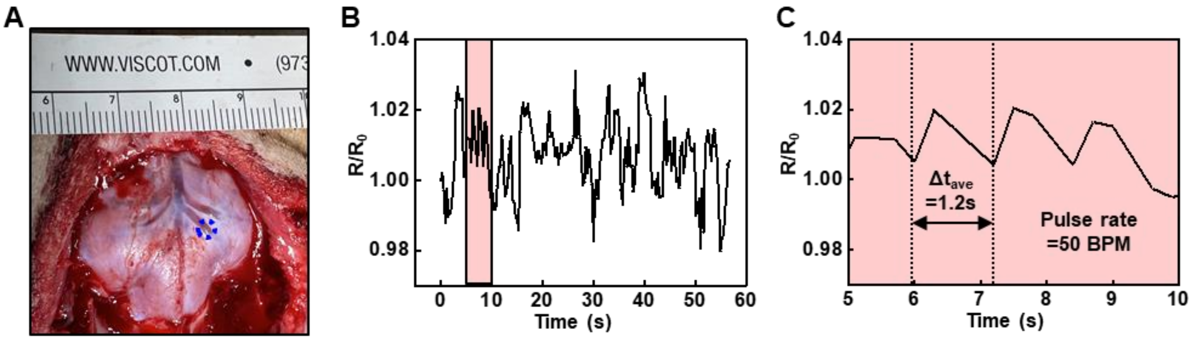

Fig. S29. *In-vivo* demonstration of cerebrovascular dynamics monitoring on a canine brain model. (**A**) Photograph of the canine brain model with the deployed position of the plane crack sensor (blue circle). (**B**) Monitoring results using the plane crack sensor on the rat brain model and (**C**) the enlarged monitoring results showing the pulse rate of 50 BPM.

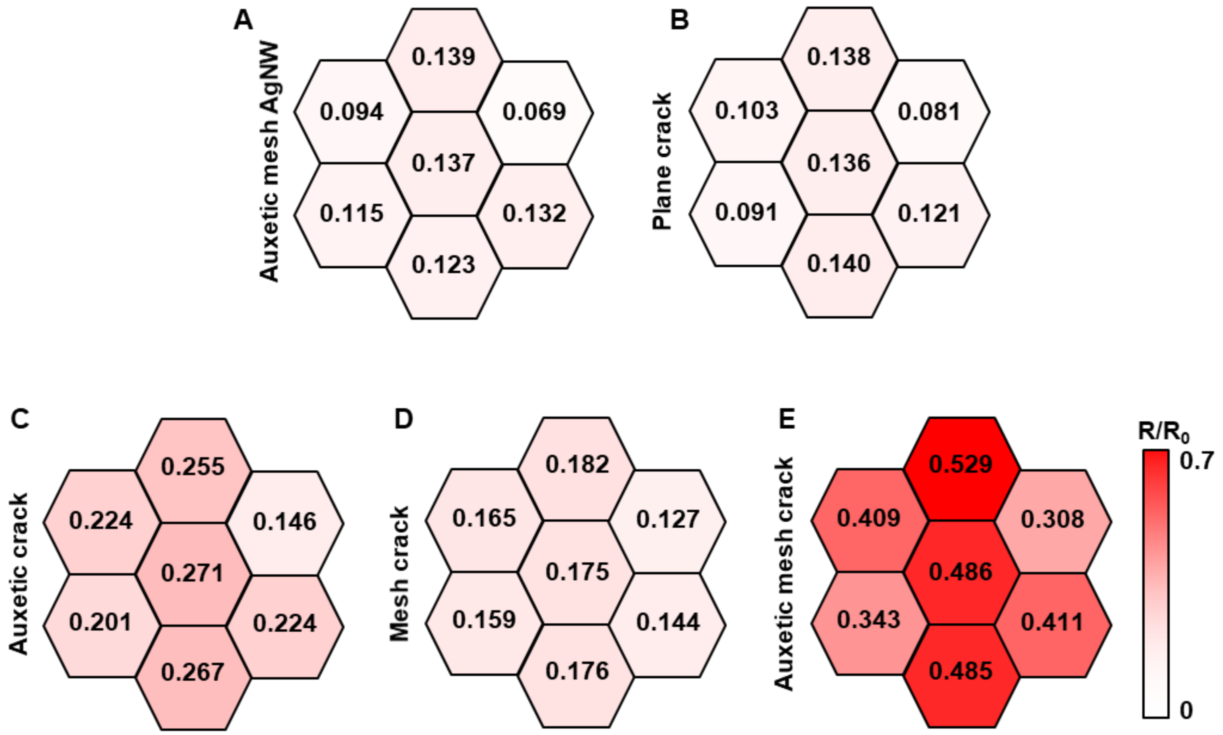

Fig. S30. Strain distribution mapping on the canine brain model based on the colorized normalized resistance variation (averaged for 10 s) with diverse type sensor array including (**A**) Ag nanowire sensors on auxetic and mesh-structured substrate, (**B**) plane crack sensors, (**C**) crack sensors on auxetic-structured substrate, (**D**) crack sensors on mesh-structured substrate, and (**E**) crack sensors on auxetic and mesh-structured substrate.

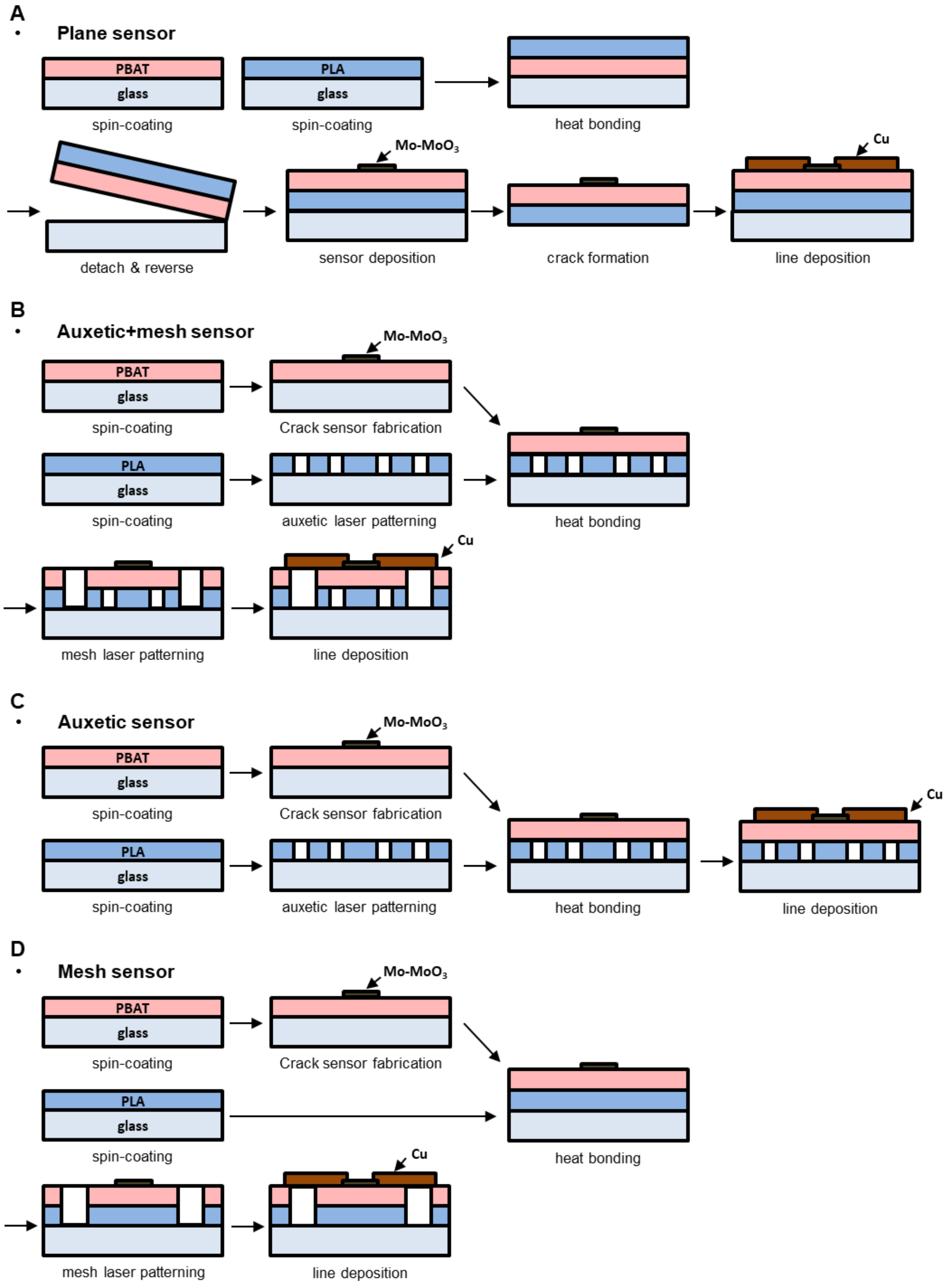

Fig. S31. Fabrication process of diverse type sensor array including (**A**) plane crack sensor, (**B**) crack sensor on auxetic and mesh-structured substrate, (**C**) crack sensor on auxetic-structured substrate, and (**D**) crack sensor on mesh-structured substrate.

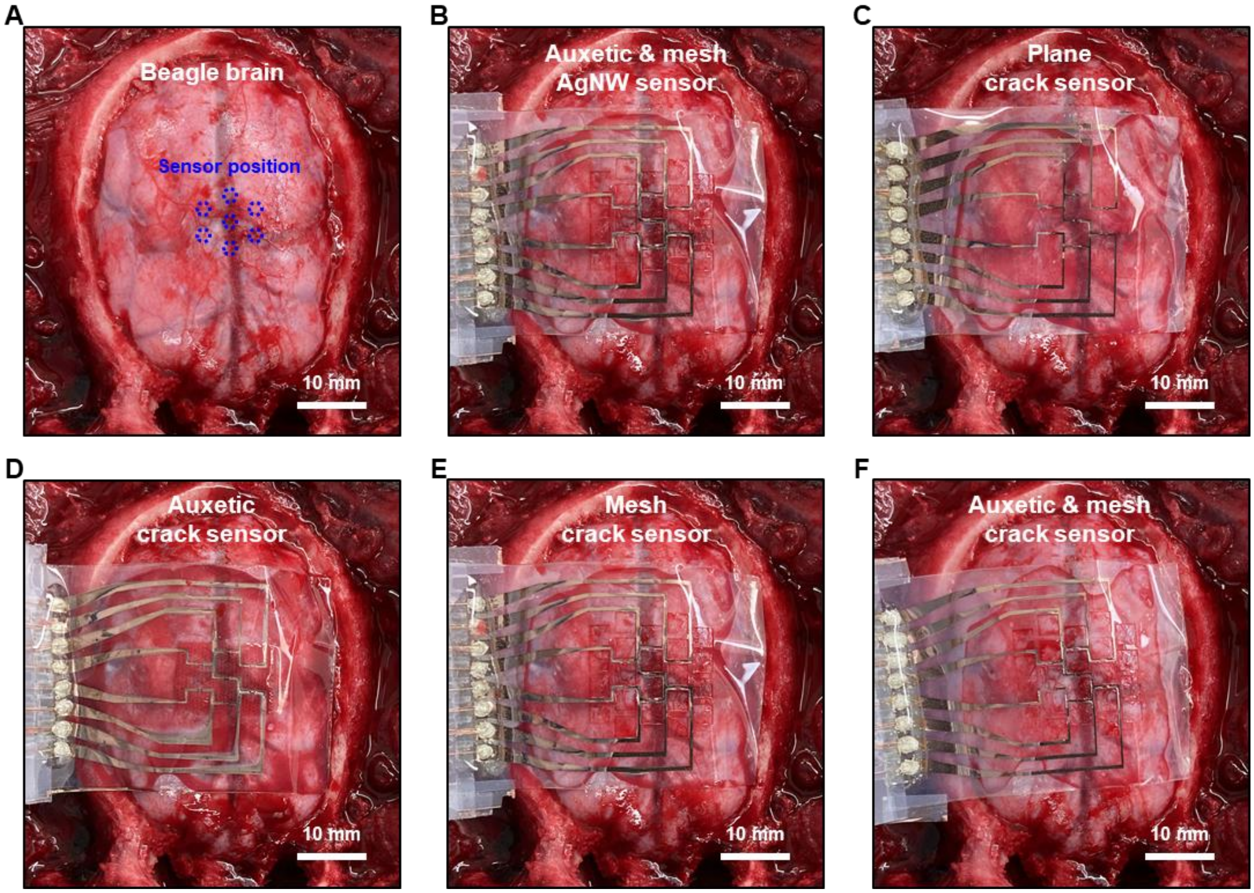

Fig. S32. Photographs of diverse type sensor array on the canine brain model. (**A**) Bare brain image with the notation of deployed locations of the sensors (blue circles). (**B**) Ag nanowire sensors on auxetic and mesh-structured substrate. (**C**) Plane crack sensors. (**D**) Crack sensors on auxetic-structured substrate. (**E**) Crack sensors on mesh-structured substrate. (**F**) Crack sensors on auxetic and mesh-structured substrate.

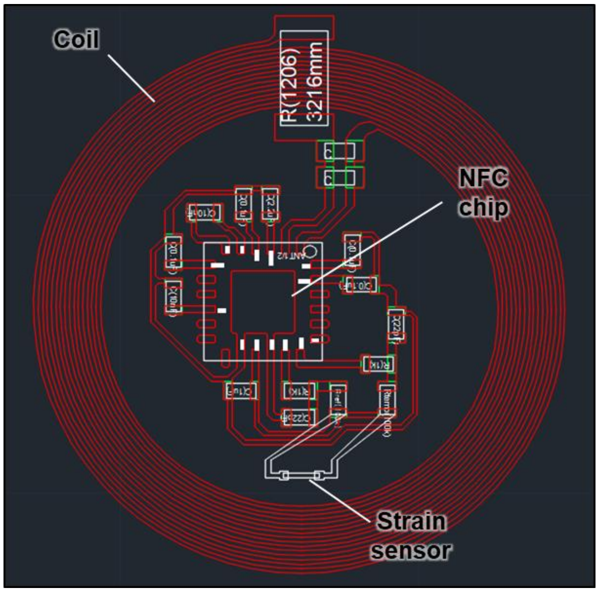

Fig. S33. CAD design image of the NFC-based wireless sensing circuit.

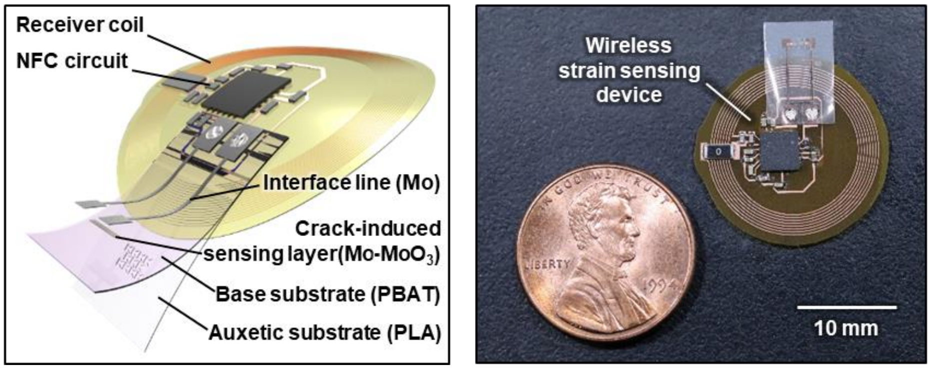

Fig. S34. Exploded view illustration (left) and photograph (right) of the meta-crack sensor integrated with NFC-based wireless sensing module, with a size comparable to a penny.

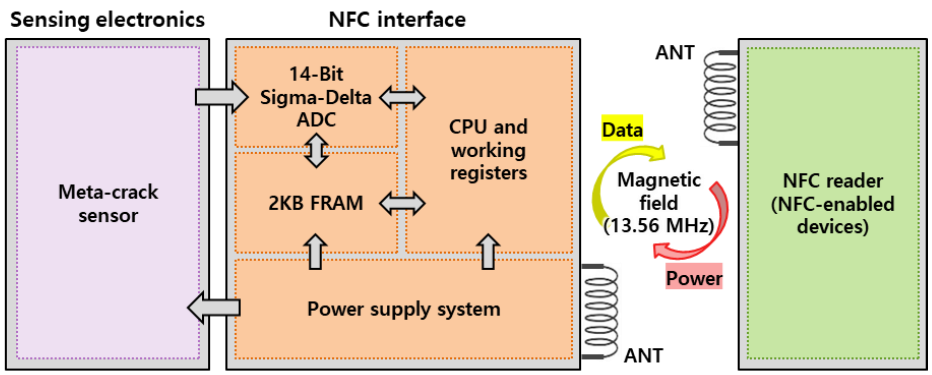

Fig. S35. Block diagram of NFC-based wireless sensing system.

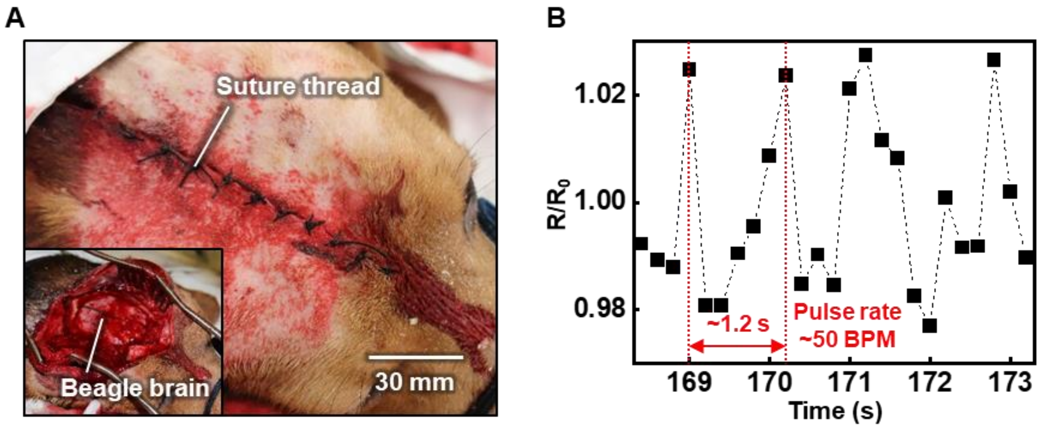

Fig. S36. *In-vivo* wireless demonstration of cerebrovascular dynamics monitoring on a canine brain model. (**A**) Photographs of the canine brain model with the implantation of the meta-crack sensor-wireless sensing module under suture state. (**B**) Monitoring results using the meta-crack sensor with NFC-based wireless sensing module on the canine brain model, showing the pulse rate of 50 BPM.

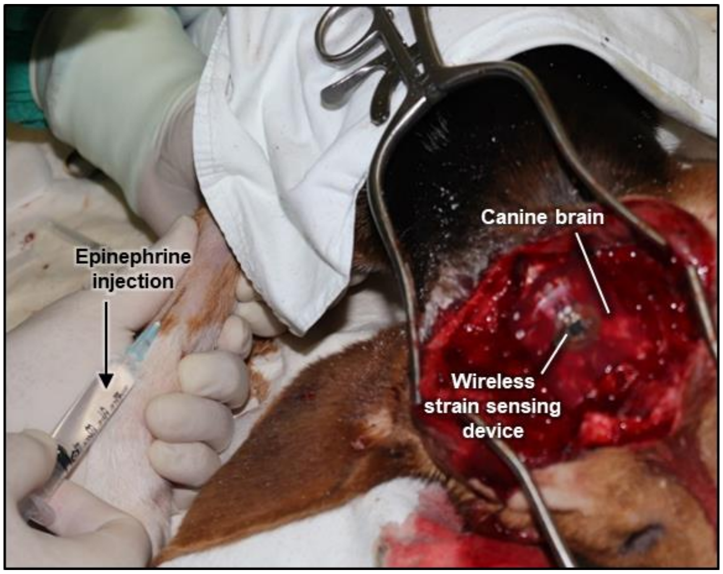

Fig. S37. Photograph of injection of epinephrine during wireless blood pressure monitoring.

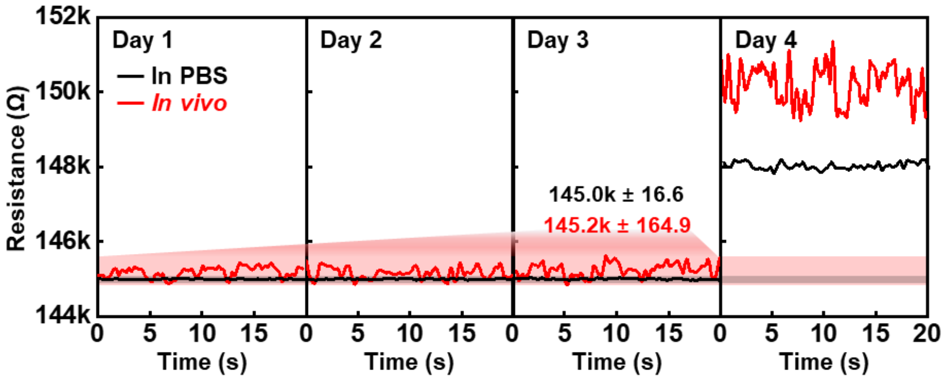

Fig. S38. Resistance variation of the sensor and the connected electrodes with encapsulation materials in PBS and on the surface of the cerebral cortex in rat brain model (*in vivo*). The base resistance showed stable variation by environmental (in PBS) and respiration (*in vivo*) noise for three days, started showing degradation from the fourth day onwards.

Materials	Gauge factor (GF)	Applied strain for the GF	Min detection limit	References
Cracked Mo / MoO_3 / PBAT	12,331	0.34%	$7.8 \cdot 10^{-5}$	This work
Cracked Au / Cr / PI	6,200	0.5%	-	Ref 29
Cracked Pt / PUA / PET	3,100	1%	-	Ref 30
Cracked Au / Cr / PI	1,300	0.5%	$1 \cdot 10^{-4}$	Ref 31
Cracked Pt / PUA	2,079	2%	-	Ref 33
Cracked AgNP / CPI	2,000	0.55%	-	Ref 47
Cracked Au / Ti / PDMS	5,000	0.7%	$6.3 \cdot 10^{-4}$	Ref 48

Table S1. Summary of key parameters of piezoresistive strain sensors reported in the literature (*29-31, 33, 47, 48*). – indicates no data were shown in the papers.

ν	k	δ_0	μ
0.3	13.54	11.71 nm	1.10408
-0.2	45.79	10.53 nm	1.54100
-0.5	97.66	11.25 nm	2.04187
-0.9	140.14	10.88 nm	1.02185

Table S2. Comparison of parameter values of theoretical modeling for the crack sensors with diverse Poisson's ratios, including measured initial crack gap size (δ_0), fitted deviation of probability distribution function (μ), and fitted proportionality factor (k).

Sensor composition	$i_{0.3,1}$	$i_{-0.5,2}$	$i_{-0.9,3}$
n:p=2:1, ν_n= -0.9	0.02	0	0.25
n:p=1:1, ν_n= -0.9	0.02	0	2.63
n:p=1:2, ν_n= -0.9	0.01	0	20.31
n:p=2:1, ν_n= -0.5	0.01	0.20	0

Table S3. Comparison of parameter values of modified theoretical modeling for the diverse parallel circuited sensors with different composition ratio of Poisson's ratios and absolute values of negative Poisson's ratio. The parameters include fitted conductance interfering factor of positive Poisson's ratio effect (ν=0.3, $i_{0.3,1}$), negative Poisson's ratio effect 1(ν=-0.5, $i_{-0.5,2}$), and negative Poisson's ratio effect 2(ν=-0.9, $i_{-0.9,3}$).

REFERENCES AND NOTES

1. A. Mongera, M. Pochitaloff, H. J. Gustafson, G. A. Stooke-Vaughan, P. Rowghanian, S. Kim, O. Campàs, Mechanics of the cellular microenvironment as probed by cells in vivo during zebrafish presomitic mesoderm differentiation. *Nat. Mater.* **22**, 135–143 (2023).

2. D. J. Shiwarski, J. W. Tashman, A. Tsamis, J. M. Bliley, M. A. Blundon, E. Aranda-Michel, Q. Jallerat, J. M. Szymanski, B. M. McCartney, A. W. Feinberg, Fibronectin-based nanomechanical biosensors to map 3D surface strains in live cells and tissue. *Nat. Commun.* **11**, 5883 (2020).

3. B. E. Danielsson, B. G. Abraham, E. Mäntylä, J. I. Cabe, C. R. Mayer, A. Rekonen, F. Ek, D. E. Conway, T. O. Ihalainen, Nuclear lamina strain states revealed by intermolecular force biosensor. *Nat. Commun.* **14**, 3867 (2023).

4. M. Chatterjee, S. Özdemir, C. Fritz, W. Möbius, L. Kleineidam, E. Mandelkow, J. Biernat, C. Doğdu, O. Peters, N. C. Cosma, X. Wang, L.-S. Schneider, J. Priller, E. Spruth, A. A. Kühn, P. Krause, T. Klockgether, I. R. Vogt, O. Kimmich, A. Spottke, D. C. Hoffmann, K. Fliessbach, C. Miklitz, C. M. Cormick, P. Weydt, B. Falkenburger, M. Brandt, R. Guenther, E. Dinter, J. Wiltfang, N. Hansen, M. Bähr, I. Zerr, A. Flöel, P. J. Nestor, E. Düzel, W. Glanz, E. Incesoy, K. Bürger, D. Janowitz, R. Perneczky, B. S. Rauchmann, F. Hopfner, O. Wagemann, J. Levin, S. Teipel, I. Kilimann, D. Goerss, J. Prudlo, T. Gasser, K. Brockmann, D. Mengel, M. Zimmermann, M. Synofzik, C. Wilke, J. Selma-González, J. Turon-Sans, Miguel Angel Santos-Santos, D. Alcolea, S. Rubio-Guerra, J. Fortea, Á. Carbayo, A. Lleó, R. Rojas-García, I. Illán-Gala, M. Wagner, I. Frommann, S. Roeske, L. Bertram, M. T. Heneka, F. Brosseron, A. Ramirez, M. Schmid, R. Beschorner, A. Halle, J. Herms, M. Neumann, N. R. Barthélemy, R. J. Bateman, P. Rizzu, P. Heutink, O. Dols-Icardo, G. Höglinger, A. Hermann, A. Schneider, Plasma extracellular vesicle tau and TDP-43 as diagnostic biomarkers in FTD and ALS. *Nat. Med.* **30**, 1771–1783 (2024).

5. L. Wang, X. Wang, Y. Wu, M. Guo, C. Gu, C. Dai, D. Kong, Y. Wang, C. Zhang, D. Qu, C. Fan, Y. Xie, Z. Zhu, Y. Liu, D. Wei, Rapid and ultrasensitive electromechanical detection of ions, biomolecules and SARS-CoV-2 RNA in unamplified samples. *Nat. Biomed. Eng.* **6**, 276–285 (2022).

6. C. Pang, G.-Y. Lee, T.-I. Kim, S. M. Kim, H. N. Kim, S.-H. Ahn, K.-Y. Suh, A flexible and highly sensitive strain-gauge sensor using reversible interlocking of nanofibres. *Nat. Mater.* **11**, 795–801 (2012).

7. C. M. Boutry, L. Beker, Y. Kaizawa, C. Vassos, H. Tran, A. C. Hinckley, R. Pfattner, S. Niu, J. Li, J. Claverie, Z. Wang, J. Chang, P. M. Fox, Z. Bao, Biodegradable and flexible arterial-pulse sensor for the wireless monitoring of blood flow. *Nat. Biomed. Eng.* **3**, 47–57 (2019).

8. C. M. Boutry, Y. Kaizawa, B. C. Schroeder, A. Chortos, A. Legrand, Z. Wang, J. Chang, P. Fox, Z. Bao, A stretchable and biodegradable strain and pressure sensor for orthopaedic application. *Nat. Electron.* **1**, 314–321 (2018).

9. O. A. Araromi, M. A. Graule, K. L. Dorsey, S. Castellanos, J. R. Foster, W.-H. Hsu, A. E. Passy, J. J. Vlassak, J. C. Weaver, C. J. Walsh, R. J. Wood, Ultra-sensitive and resilient compliant strain gauges for soft machines. *Nature* **587**, 219–224 (2020).

10. S. Liu, J. Zhang, Y. Zhang, R. Zhu, A wearable motion capture device able to detect dynamic motion of human limbs. *Nat. Commun.* **11**, 5615 (2020).

11. G. Valle, N. Katic Secerovic, D. Eggemann, O. Gorskii, N. Pavlova, F. M. Petrini, P. Cvancara, T. Stieglitz, P. Musienko, M. Bumbasirevic, S. Raspopovic, Biomimetic computer-to-brain communication enhancing naturalistic touch sensations via peripheral nerve stimulation. *Nat. Commun.* **15**, 1151 (2024).

12. M. C. Benn, S. A. Pot, J. Moeller, T. Yamashita, C. M. Fonta, G. Orend, P. Kollmannsberger, V. Vogel, How the mechanobiology orchestrates the iterative and reciprocal ECM-cell cross-talk that drives microtissue growth. *Sci. Adv.* **9**, eadd9275 (2023).

13. N. Marina, I. N. Christie, A. Korsak, M. Doronin, A. Brazhe, P. S. Hosford, J. A. Wells, S. Sheikhbahaei, I. Humoud, J. F. R. Paton, M. F. Lythgoe, A. Semyanov, S. Kasparov, A. V. Gourine, Astrocytes monitor cerebral perfusion and control systemic circulation to maintain brain blood flow. *Nat. Commun.* **11**, 131 (2020).

14. A. Senoussi, J.-C. Galas, A. Estevez-Torres, Programmed mechano-chemical coupling in reaction-diffusion active matter. *Sci. Adv.* **7**, eabi9865 (2021).

15. F. J. Armistead, J. Gala De Pablo, H. Gadêlha, S. A. Peyman, S. D. Evans, Physical biomarkers of disease progression: On-chip monitoring of changes in mechanobiology of colorectal cancer cells. *Sci. Rep.* **10**, 3254 (2020).

16. A. Abramson, C. T. Chan, Y. Khan, A. Mermin-Bunnell, N. Matsuhisa, R. Fong, R. Shad, W. Hiesinger, P. Mallick, S. S. Gambhir, Z. Bao, A flexible electronic strain sensor for the real-time monitoring of tumor regression. *Sci. Adv.* **8**, eabn6550 (2022).

17. M. Plodinec, M. Loparic, C. A. Monnier, E. C. Obermann, R. Zanetti-Dallenbach, P. Oertle, J. T. Hyotyla, U. Aebi, M. Bentires-Alj, R. Y. H. Lim, C.-A. Schoenenberger, The nanomechanical signature of breast cancer. *Nat. Nanotechnol.* **7**, 757–765 (2012).

18. J.-Y. Yoo, S. Oh, W. Shalish, W.-Y. Maeng, E. Cerier, E. Jeanne, M.-K. Chung, S. Lv, Y. Wu, S. Yoo, A. Tzavelis, J. Trueb, M. Park, H. Jeong, E. Okunzuwa, S. Smilkova, G. Kim, J. Kim, G. Chung, Y. Park, A. Banks, S. Xu, G. M. Sant'Anna, D. E. Weese-Mayer, A. Bharat, J. A. Rogers, Wireless broadband acousto-mechanical sensing system for continuous physiological monitoring. *Nat. Med.* **29**, 3137–3148 (2023).

19. Y. J. Kang, H. M. Arafa, J.-Y. Yoo, C. Kantarcigil, J.-T. Kim, H. Jeong, S. Yoo, S. Oh, J. Kim, C. Wu, A. Tzavelis, Y. Wu, K. Kwon, J. Winograd, S. Xu, B. Martin-Harris, J. A. Rogers, Soft skin-interfaced mechano-acoustic sensors for real-time monitoring and patient feedback on respiratory and swallowing biomechanics. *npj Digit. Med.* **5**, 147 (2022).

20. S.-K. Kang, R. K. J. Murphy, S.-W. Hwang, S. M. Lee, D. V. Harburg, N. A. Krueger, J. Shin, P. Gamble, H. Cheng, S. Yu, Z. Liu, J. G. McCall, M. Stephen, H. Ying, J. Kim, G. Park, R. C. Webb, C. H. Lee, S. Chung, D. S. Wie, A. D. Gujar, B. Vemulapalli, A. H. Kim, K.-M. Lee, J. Cheng, Y. Huang, S. H. Lee, P. V. Braun, W. Z. Ray, J. A. Rogers, Bioresorbable silicon electronic sensors for the brain. *Nature* **530**, 71–76 (2016).

21. T. Kim, Y. Shin, K. Kang, K. Kim, G. Kim, Y. Byeon, H. Kim, Y. Gao, J. R. Lee, G. Son, T. Kim, Y. Jun, J. Kim, J. Lee, S. Um, Y. Kwon, B. G. Son, M. Cho, M. Sang, J. Shin, K. Kim, J. Suh, H. Choi, S. Hong, H. Cheng, H.-G. Kang, D. Hwang, K. J. Yu, Ultrathin crystalline-silicon-based strain gauges with deep learning algorithms for silent speech interfaces. *Nat. Commun.* **13**, 5815 (2022).

22. J. Guo, B. Zhou, R. Zong, L. Pan, X. Li, X. Yu, C. Yang, L. Kong, Q. Dai, Stretchable and highly sensitive optical strain sensors for human-activity monitoring and healthcare. *ACS Appl. Mater. Interfaces* **11**, 33589–33598 (2019).

23. J. Guo, M. Niu, C. Yang, Highly flexible and stretchable optical strain sensing for human motion detection. *Optica* **4**, 1285–1288 (2017).

24. X. Guo, W. Hong, T. Zhang, H. Li, T. Zhu, Q. Hong, D. Wang, L. Liu, Y. Zhao, D. Wang, Z. Mai, M. Wang, F. Yan, Y. Meng, Y. Xu, G. Xing, Highly stretchable, responsive flexible dual-mode magnetic strain sensor. *Adv. Mater. Technol.* **8**, 2201439 (2023).

25. Q. Zhang, G. Yang, L. Xue, G. Dong, W. Su, M. J. Cui, Z. G. Wang, M. Liu, Z. Zhou, X. Zhang, Ultrasoft and biocompatible magnetic-hydrogel-based strain sensors for wireless passive biomechanical monitoring. *ACS Nano* **16**, 21555–21564 (2022).

26. J. J. Sloots, G. J. Biessels, A. de Luca, J. J. M. Zwanenburg, Strain tensor imaging: Cardiac-induced brain tissue deformation in humans quantified with high-field MRI. *Neuroimage* **236**, 118078 (2021).

27. J.-Y. Bae, G.-S. Hwang, Y.-S. Kim, J. Jeon, M. Chae, J.-W. Kim, S. Lee, S. Kim, S.-H. Lee, S.-G. Choi, J.-Y. Lee, J.-H. Lee, K.-S. Kim, J.-H. Park, W.-J. Lee, Y.-C. Kim, K.-S. Lee, J. Kim, H. Lee, J. K. Hyun, J.-Y. Kim, S.-K. Kang, A biodegradable and self-deployable electronic tent electrode for brain cortex interfacing. *Nat. Electron.* **7**, 815–828 (2024).

28. D.-H. Kim, J. Viventi, J. J. Amsden, J. Xiao, L. Vigeland, Y.-S. Kim, J. A. Blanco, B. Panilaitis, E. S. Frechette, D. Contreras, D. L. Kaplan, F. G. Omenetto, Y. Huang, K.-C. Hwang, M. R.

Zakin, B. Litt, J. A. Rogers, Dissolvable films of silk fibroin for ultrathin conformal bio-integrated electronics. *Nat. Mater.* **9**, 511–517 (2010).

29. Y. Roh, M. Kim, S. M. Won, D. Lim, I. Hong, S. Lee, T. Kim, C. Kim, D. Lee, S. Im, G. Lee, D. Kim, D. Shin, D. Gong, B. Kim, S. Kim, S. Kim, H. K. Kim, B.-K. Koo, S. Seo, J.-S. Koh, D. Kang, S. Han, Vital signal sensing and manipulation of a microscale organ with a multifunctional soft gripper. *Sci. Robot.* **6**, eabi6774 (2021).

30. B. Park, J. Kim, D. Kang, C. Jeong, K. S. Kim, J. U. Kim, P. J. Yoo, T.-i. Kim, Dramatically enhanced mechanosensitivity and signal-to-noise ratio of nanoscale crack-based sensors: Effect of crack depth. *Adv. Mater.* **28**, 8130–8137 (2016).

31. T. Kim, I. Hong, Y. Roh, D. Kim, S. Kim, S. Im, C. Kim, K. Jang, S. Kim, M. Kim, J. Park, D. Gong, K. Ahn, J. Lee, G. Lee, H.-S. Lee, J. Kang, J. M. Hong, S. Lee, S. Seo, B.-K. Koo, J.-S. Koh, S. Han, D. Kang, Spider-inspired tunable mechanosensor for biomedical applications. *npj Flex Electron.* **7**, 12 (2023).

32. Y. Huang, Y. Xiang, W. Ren, F. Li, C. Li, T. Yang, Enhancing the sensitivity of crack-based strain sensor assembled by functionalized graphene for human motion detection. *Sci. China Technol. Sci.* **64**, 1805–1813 (2021).

33. D. Kang, P. V. Pikhitsa, Y. W. Choi, C. Lee, S. S. Shin, L. Piao, B. Park, K.-Y. Suh, T.-I. Kim, M. Choi, Ultrasensitive mechanical crack-based sensor inspired by the spider sensory system. *Nature* **516**, 222–226 (2014).

34. T. Sakorikar, M. K. Kavitha, P. Vayalamkuzhi, M. Jaiswal, Thickness-dependent crack propagation in uniaxially strained conducting graphene oxide films on flexible substrates. *Sci. Rep.* **7**, 2598 (2017).

35. J.-H. Lee, J.-Y. Bae, Y.-N. Kim, M. Chae, W.-J. Lee, J. Lee, I.-D. Kim, J. K. Hyun, K.-S. Lee, D. Kang, S.-K. Kang, A fully biodegradable and ultra-sensitive crack-based strain sensor for biomechanical signal monitoring. *Adv. Funct. Mater.* **34**, 2406035 (2024).

36. E. Lee, T. Kim, H. Suh, M. Kim, P. V. Pikhitsa, S. Han, J.-S. Koh, D. Kang, Effect of metal thickness on the sensitivity of crack-based sensors. *Sens.* **18**, 2872 (2018).

37. W. B. Han, G.-J. Ko, K.-G. Lee, D. Kim, J. H. Lee, S. M. Yang, D.-J. Kim, J.-W. Shin, T.-M. Jang, S. Han, H. Zhou, H. Kang, J. H. Lim, K. Rajaram, H. Cheng, Y.-D. Park, S. H. Kim, S.-W. Hwang, Ultra-stretchable and biodegradable elastomers for soft, transient electronics. *Nat. Commun.* **14**, 2263 (2023).

38. W. Hwang, J. Kim, S. Park, T.-H. Kang, S. Kim, K. Lee, M.-G. Lee, R. Kwak, I.-S. Choi, H. Yi, A breathable and stretchable metastructure for a versatile hybrid electronic skin patch with long-term skin comfort. *Adv. Mater. Technol.* **8**, 2200477 (2023).

39. Y. Wang, S. Lee, T. Yokota, H. Wang, Z. Jiang, J. Wang, M. Koizumi, T. Someya, A durable nanomesh on-skin strain gauge for natural skin motion monitoring with minimum mechanical constraints. *Sci. Adv.* **6**, eabb7043 (2020).

40. Y. Jiang, Z. Liu, N. Matsuhisa, D. Qi, W. R. Leow, H. Yang, J. Yu, G. Chen, Y. Liu, C. Wan, Z. Liu, X. Chen, Auxetic mechanical metamaterials to enhance sensitivity of stretchable strain sensors. *Adv. Mater.* **30**, 1706589 (2018).

41. L. E. Hoffman, L. H. S. Van Mierop, Effect of epinephrine on heart rate and arterial blood pressure of the developing chick embryo. *Pediatr. Res.* **5**, 472–477 (1971).

42. J. Wu, D.-S. Zhang, M.-H. Ji, Z. Zhang, J.-J. Yang, Hemodynamic effects of epinephrine in healthy and hemorrhagic shock rats. *Curr. Ther. Res.* **72**, 243–249 (2011).

43. X. Shi, J. Yu, Q. Pan, Y. Lu, L. Li, H. Cao, Impact of total epinephrine dose on long term neurological outcome for cardiac arrest patients: A cohort study. *Front. Pharmacol.* **12**, 580234 (2021).

44. T. Kim, I. Hong, M. Kim, S. Im, Y. Roh, C. Kim, J. Lim, D. Kim, J. Park, S. Lee, D. Lim, J. Cho, S. Huh, S.-U. Jo, C. Kim, J.-S. Koh, S. Han, D. Kang, Ultra-stable and tough bioinspired crack-based tactile sensor for small legged robots. *npj Flex Electron.* **7**, 22 (2023).

45. T. Pepelnjak, A. Karimi, A. Maček, N. Mole, Altering the elastic properties of 3D printed poly-lactic acid (PLA) parts by compressive cyclic loading. *Materials* **13**, 4456 (2020).

46. N. Perumal, S. Sreekantan, Z. A. A. Hamid, A. Rusli, K. Bhubalan, J. N. Appaturi, Effect of plasticizer and compatibilizer on properties of polybutylene adipate-co-terephthalate (PBAT) with acetylated starch. *J. Polym. Environ.* **32**, 289–302 (2024).

47. K. K. Kim, I. Ha, M. Kim, J. Choi, P. Won, S. Jo, S. H. Ko, A deep-learned skin sensor decoding the epicentral human motions. *Nat. Commun.* **11**, 2149 (2020).

48. T. Yang, X. Li, X. Jiang, S. Lin, J. Lao, J. Shi, Z. Zhen, Z. Li, H. Zhu, Structural engineering of gold thin films with channel cracks for ultrasensitive strain sensing. *Mater. Horiz.* **3**, 248–255 (2016).
